# The Effect of Combining mHealth and Health Professional–Led Intervention for Improving Health-Related Outcomes in Chronic Diseases: Systematic Review and Meta-Analysis

**DOI:** 10.2196/55835

**Published:** 2025-01-20

**Authors:** Masashi Kanai, Takahiro Miki, Toshiya Sakoda, Yuta Hagiwara

**Affiliations:** 1 PREVENT Inc Aichi Japan; 2 Institute of Transdisciplinary Science for Innovation Kanawaza University Kanazawa Japan

**Keywords:** mHealth, systematic reviews, meta-analysis, chronic diseases, global health, technology, health care, interventions, chronic conditions, health care professionals, World Health Organization, physical activity, web-based

## Abstract

**Background:**

Chronic diseases such as diabetes and cardiovascular disease are global health challenges, affecting millions of people worldwide. Traditional health care often falls short in chronic disease management. This has led to the exploration of innovative solutions, such as mobile health (mHealth) technologies. mHealth, which leverages mobile and wireless technologies, has the potential to transform health care delivery by providing continuous, accessible, and personalized care. However, the effectiveness of mHealth, particularly when integrated with traditional health care interventions delivered by professionals, warrants comprehensive investigation. Understanding the combined impact of mHealth and professional-led interventions is critical to maximizing the potential of mHealth to improve patient outcomes and adherence.

**Objective:**

This study aims to investigate the effectiveness of combining mHealth and health professional–led intervention for improving health-related outcomes in chronic diseases

**Methods:**

This systematic review and meta-analysis focused on randomized controlled trials. We searched Web of Science, CENTRAL, MEDLINE, and CINAHL through July 17, 2023. The study targeted patients aged 18 years and older, experiencing at least 1 chronic condition. The interventions were a combination of mHealth and the use of a health care professional. The comparison groups consisted of participants receiving either general care and follow-up or those using mHealth devices without any health care professional involvement. The outcomes measured in this review included hemoglobin A_1c_ (HbA_1c_), quality of life (QoL), and physical activity.

**Results:**

The study included 26 research papers, encompassing 7360 individuals. Meta-analysis was conducted for HbA_1c_, QoL, and physical activity. For HbA_1c_, short-term improvement was significant (standardized mean difference [SMD] –0.43; 95% CI –0.64 to –0.21; *I*^2^=69%) and medium term (SMD –0.49; 95% CI –0.49 to –0.09; *I*^2^=21%). However, in the long term, the improvement was not significant (SMD –0.23; 95% CI –0.49 to 0.03; *I*^2^=88%). For QoL, significant improvements were observed in the short term (SMD –0.23; 95% CI –0.42 to –0.05; *I*^2^=62%), and in the medium term (SMD –0.16; 95% CI –0.24 to –0.07; *I*^2^=0%). In the long term, however, the improvement was not significant (SMD –0.12; 95% CI –0.41 to 0.16; *I*^2^=71%). For physical activity, both subjective (questionnaire) and objective (number of steps) outcomes were analyzed. In the short term, subjective outcomes showed significant improvement (SMD 0.31; 95% CI 0.12-0.50; *I*^2^=0%), while objective outcomes did not (SMD 0.11; 95% CI –0.05 to 0.27; *I*^2^=0%). Medium- and long-term subjective outcomes showed no significant improvement. Meta-analysis for objective outcomes in the medium and long term was not possible due to insufficient studies.

**Conclusions:**

This study confirmed short- and medium-term benefits of mHealth combined with professional interventions for HbA_1c_, QoL, and short-term physical activity, supporting effective chronic disease management.

## Introduction

### Background

Mobile health (mHealth) is the delivery of medical and public health services using mobile handsets, mobile kiosks, and other wireless terminals in real-world environments [1]. Interventions in mHealth can take various forms, including the creation of original apps for the management of health conditions and communication, simple text message-based interventions, as well as those using voice calls or the accumulation of device data on mobile apps for data management [2]. Many interventions in the field of mHealth primarily emphasize self-management without any direct human involvement [3]. Likewise, there are interventions that involve periodic health care professional assistance and the additional use of health devices [4,5].

The growing disease burden of chronic diseases, on the other hand, is one of the global challenges [6]. Chronic diseases include conditions such as cardiovascular diseases, stroke, cancer, diabetes, respiratory conditions, and arthritis [7]. Furthermore, obesity is now defined as a disease, and the most common chronic disease [8]. While the assistance of health care professionals is essential for the management of these chronic conditions, mHealth may be valuable in filling the gaps between medical consultations or support, as well as in the monitoring of disease conditions remotely. In fact, the efficacy of therapeutic apps prescribed by physicians has also been reported [9]. The effectiveness of mHealth is currently being investigated through a systematic review [10]. The quality of research methodologies is often limited, and consistent conclusions have not been reached yet. Among the reasons for this is that the definition of mHealth intervention methods varies widely, and depending on the definition, conclusions about effectiveness can differ. Some reports suggest that no significant effects have been observed. There is a discrepancy in research on health interventions that focus on self-monitoring for chronic diseases, and it is unclear if there are any conclusive benefits [11-13]. A systematic review evaluating the effectiveness of remote monitoring in various types of chronic diseases found only limited improvements in general health status and clinical outcomes [14].

From a behavioral science perspective, effective management of people with chronic conditions requires more than just the use of mHealth technologies. It also requires consistent support from health care professionals over medium and long terms. In particular, studies focused on obesity have used interaction with professionals as an indicator of engagement. This interaction has been shown to play a significant role in achieving long-term (ie, 1 year) weight loss outcomes [15] although these definitions may vary between studies, potentially affecting the interpretation of results. Therefore, to maximize the potential of mHealth, we hypothesized that combining mHealth with health professional–led interventions would be more effective than relying solely on health care use. To investigate this hypothesis, our study focused exclusively on interventions that integrated mHealth with professional-led chronic disease strategies and then evaluated their effectiveness for each different duration.

### Objectives

With this systematic review and meta-analysis, we investigated the effectiveness of combining mHealth and health professional–led intervention for improving health-related outcomes in chronic diseases.

## Methods

### Study Design

This was a systematic review and meta-analysis of randomized controlled trials.

### Protocol and Registration

This systematic review was preregistered in the PROSPERO database (CRD42022337882) and conducted following the PRISMA (Preferred Reporting Items for Systematic Reviews and Meta-Analyses) guidelines (Multimedia Appendix 1).

### Search Strategy

Our search spanned 4 databases from their start dates until July 17, 2023. These included Web of Science, CENTRAL, MEDLINE, and CINAHL, as they are the four most used databases in similar research. Manual searches systematically screened the reference lists of all studies identified in the database search. The same inclusion criteria were applied to these references and the titles and abstracts were reviewed to determine whether they were included studies. Our systematic search strategy used both medical subject heading terms and keywords derived from subject headings. We limited our inclusion to randomized controlled trials published in English, excluding gray literature. The detailed search strategy is available in Multimedia Appendix 2.

### Identification and Selection of Trials

#### Population

The population of interest included patients aged 18 years and older with at least 1 chronic condition. This was a change from the original protocol (CRD42022337882), which specified a minimum age of 20 years, in order to increase the generalizability of the findings by including all adults.

#### Interventions

This study’s interventions comprised a mix of mHealth and direct health care professional involvement. The World Health Organization defines mHealth as “the use of mobile and wireless technologies to support health objectives” [16]. We excluded interventions that primarily used computers, categorizing them as eHealth. Additionally, interventions where participants used smartphones solely for calls or text messages, without using specific apps, were not included to avoid overlapping mHealth and eHealth interventions and to ensure a clear distinction. The health care professionals involved in our study spanned various fields, including doctors, nurses, physiotherapists, occupational therapists, and dieticians. These professionals were actively engaged in delivering the mHealth intervention, beyond just explaining or reviewing app data. The methods used by health professionals for interventions included face-to-face interactions, phone calls, and text messaging.

#### Comparison

The comparison groups in this study consisted of the general control groups used in each individual study. For example, control groups were defined as those with only general care and follow-up, or those using mHealth-related devices but no involvement of a health care professional.

#### Outcomes

This systematic review assessed the following primary outcomes: HbA_1c_ levels, quality of life (QoL), and physical activity. These outcomes were chosen for their relevance in chronic disease management and were analyzed across different follow-up periods (short, medium, and long terms) to evaluate intervention effectiveness over time.

#### Selection Process

Rayyan software was used. Two authors (MK and TS) independently assessed the title and abstract records to identify eligible studies. The same authors then conducted a full-text screening to assess whether the criteria were met; in cases where the 2 authors’ judgments conflicted, a third author (TM) decided and ultimately reached a consensus.

#### Assessment of Methodological Quality

The risk of bias for individual studies was assessed using the Cochrane Risk of Bias Framework for sequence generation, allocation concealment, blinding, completeness of outcome assessment, selective reporting, and other biases. Data were extracted by 2 authors (TM and MK). Disagreements were reviewed by a third author and a final consensus was reached. For the quality of the clinical evidence, the GRADE (Grading of Recommendations Assessment, Development, and Evaluation) system was used to evaluate the results.

### Data Extraction

Data were extracted by 2 authors (TM and MK). Disagreements were reviewed by a third reviewer and a final consensus was reached. Data were extracted on (1) basic information about the study, such as authors, year of publication, country, and region where the intervention took place; (2) target population; (3) intervention group description; (4) control group description; (5) outcomes; and (6) key findings.

### Data Analysis

When two or more studies assessed the same outcome, a meta-analysis was performed using Review Manager 5.4 (Nordic Cochrane Centre). The results of the intervention groups were compared with the control group in each meta-analysis. The combined app and professional intervention was considered the intervention group and compared to the control group when there were more than 2 groups. The meta-analysis evaluated 3 different categories of time periods: short-, medium-, and long-term effects. Short term was defined as the closest to 3 months, medium term as the closest to 6 months, and long term as the closest to 12 months. For each follow-up point, the mean and SD were calculated. The group mean was calculated from the SE, CI, and *P* value of the difference between the means in trials that did not report SDs for each intervention group. In studies that only reported the median and IQR without providing SDs, we estimated the mean using the median value directly and approximated the SD by multiplying the IQR by 1.35. This method follows established meta-analytic conventions for handling summary statistics in studies with limited data [17]. All meta-analyses were presented as standardized mean difference (SMD) to account for adjusted and unadjusted means, due to the different data presentation formats of the included studies [17]. The authors of the studies in which the data were presented in a graphical format were contacted to obtain the exact values. If the reporting of results was still unclear after the above procedure, studies were excluded from the meta-analysis. For each meta-analysis, heterogeneity was assessed using *I*². When the *I*² value exceeded 75%, sensitivity analysis was conducted by sequentially excluding studies with the largest effect sizes, as these studies contributed significantly to the overall heterogeneity. This stepwise exclusion continued until heterogeneity dropped below the 75% threshold. By reducing the influence of studies with disproportionately high effect sizes, we aimed to create a more consistent and interpretable dataset, enhancing the reliability of the pooled effect size and minimizing the risk of bias or misinterpretation due to extreme variability among studies [18].

## Results

### Study Selection

**Figure 1:**
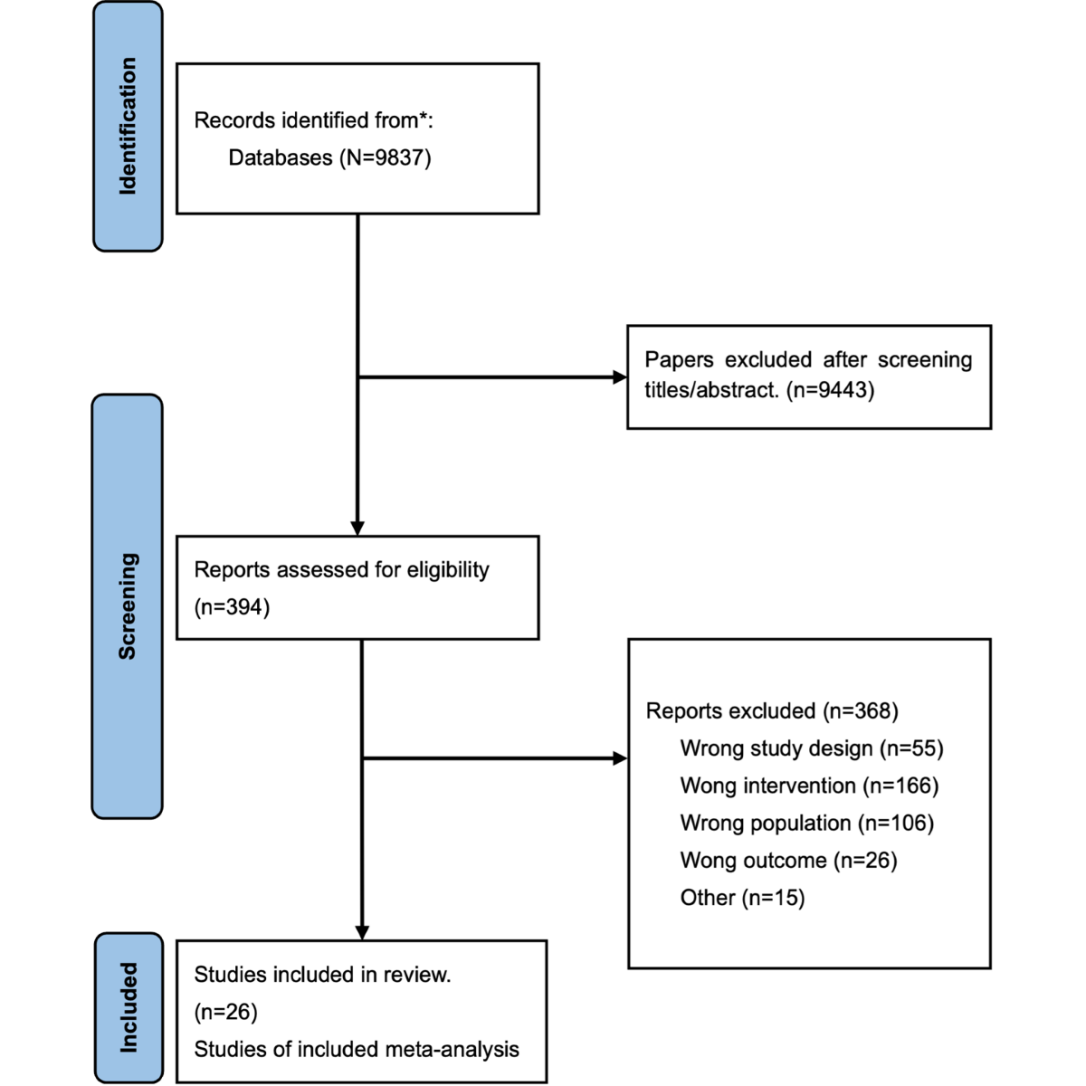
Flow diagram.

The initial search identified 9837 records, which were screened for title and text as primary screening after removing duplicates. A number of 394 were selected for secondary screening after the first screening. Finally, 26 studies were finally selected. Figure 1 shows the flow chart of the selection process.

### Characteristics of Included Trials and Intervention Description

Regarding the country, studies were conducted at outpatient or community practices in the following countries: Pakistan (2/26, 8%) [19,20], Spain (4/26, 15%) [21-24], Mexico (2/26, 8%) [25,26], United Kingdom (4/26, 15%) [27-30], United States (2/26, 8%) [31,32], China (4/26, 15%) [33-36], Singapore (2/26, 8%) [34,37]; and one each in Netherlands [38], Australia [39], Canada [40], Germany [41], Indonesia [42], and Thailand [43].

Regarding the disease, diabetes was the most common disease, with 12 of 26 trials [21,22,25,29-31,33,34,36,39,42,43]. Seven studies included type 2 diabetes [21,25,30,31,33,34,39] and 5 studies included both [22,29,36,42,43]. No study included only type 1 diabetes. The next most common was heart disease, which accounted for 7 of the 26 cases [19,20,24,35,37,38,44]. Other disease areas included cancer (2 studies) [26,32], respiratory diseases (1 study) [28], chronic diseases (2 studies) [23,40], metabolic syndrome (1 study) [41], and depression (1 study) [27].

The most common outcome was quality of life. There were 15 studies [20,22,24-27,30,32,34,37-41,44]. Ten studies used physical activity as an outcome [19,21,26,28,30,32,35,39,40,44]. Details are provided in Multimedia Appendix 3 [19-44].

### Risk of Bias Assessment

The results of the risk of bias assessment are summarized in Multimedia Appendix 4 [19-44]. The risk of bias assessment for studies on HbA_1c_, QoL, and physical activity (steps and subjective measures) showed that most studies had a low risk of bias. For example, studies like Anzaldo-Campos et al [25] and Blair et al [26] were evaluated as low-risk. Some studies had some concerns, such as Azelton et al [31] and Gill et al [40], but none were classified as high risk. Several studies, including Franc et al [22] and Manzoor et al [19], did not have sufficient data for a full assessment.

### Certainty of the Evidence According to the GRADE Approach

The certainty of evidence results using the GRADE approach are described in the results for each outcome. The GRADE assessment showed varying levels of evidence quality across outcomes. Most HbA_1c_ outcomes had very low to moderate certainty, with short-term outcomes showing very low certainty and medium-term outcomes showing moderate certainty. QoL outcomes generally had low to moderate certainty, with long-term outcomes showing very low certainty. Physical activity outcomes varied, with step counts showing moderate certainty and subjective measures ranging from very low to moderate certainty. These findings highlight the need for caution due to risks of bias and inconsistencies. A table with the results is provided in Multimedia Appendix 5.

### Synthesis of Results: Meta-Analysis

One study was excluded because data were not available [24]. Therefore, a meta-analysis was performed from 25 studies. Our systematic review included a broad spectrum of conditions, but diabetes was the most frequently addressed condition among the included studies. Consequently, HbA_1c_ was chosen as a primary indicator due to its importance in diabetes management and its frequent reporting in the studies. QoL was selected as it is a critical outcome measure reflecting the overall well-being of patients with chronic conditions. Physical activity and subjective outcomes of physical activity were included because these measures are commonly used to assess lifestyle modifications and their impact on chronic disease management. These indicators were the most frequently reported across the studies we reviewed, making them the most relevant for our analysis.

### HbA_1c_ Outcome

#### Short Term

**Figure 2:**
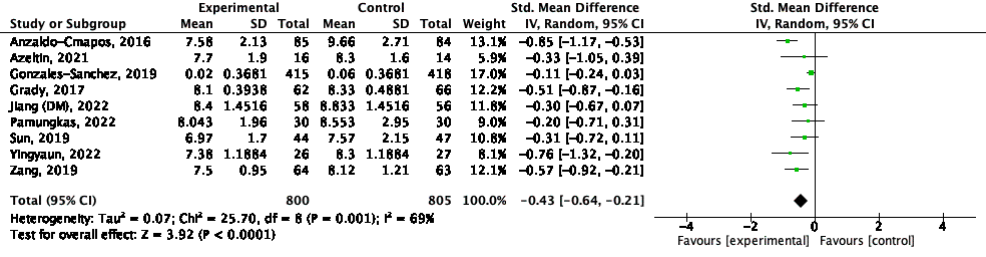
Hemoglobin A1c (HbA_1c_) for short term [23,25,29,31,33,34,36,42,43].

Meta-analysis was performed on 9 studies [23,25,29,31,33,34,36,42,43]. There was a significant increase in improvement for the combination intervention (SMD –0.43; 95% CI –0.64 to –0.21; *P*<.001; *I*^2^=69%; Figure 2 [23,25,29,31,33,34,36,42,43]).

#### Medium Term

**Figure 3:**
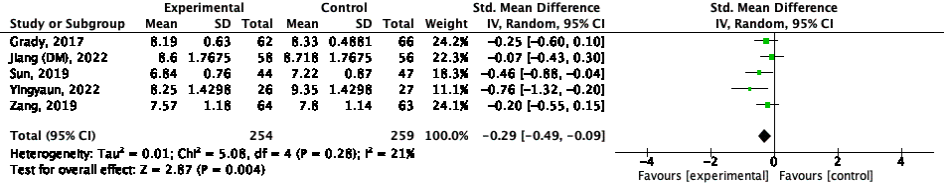
Hemoglobin A1c (HbA_1c_) for medium term [29,33,34,36,43].

A meta-analysis was performed on 5 studies [29,33,34,36,43]. There was a significant increase in improvement for the combination intervention (SMD –0.49; 95% CI –0.49 to –0.09; *P*<.001; *I*^2^=21%; Figure 3 [29,33,34,36,43]).

#### Long Term

**Figure 4:**
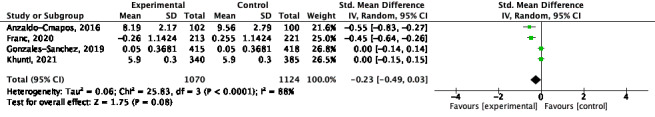
Hemoglobin A1c (HbA_1c_) for long term [22,23,25,30].

One study was excluded because data were not available [24]. A meta-analysis was performed on 4 studies [22,23,25,30]. There was no significant increase in improvement for the combination intervention in the long term (SMD –0.23; 95% CI –0.49 to 0.03; *P*<.001; *I*^2^=88%; Figure 4 [22,23,25,30]). Sensitivity analysis was performed because heterogeneity was >75%. Two studies [23,30] were excluded and did not reach statistical significance (SMD –0.00; 95% CI –0.10 to 0.10; *P*<.001; *I*^2^=0%).

### QoL Outcome

#### Short Term

**Figure 5:**
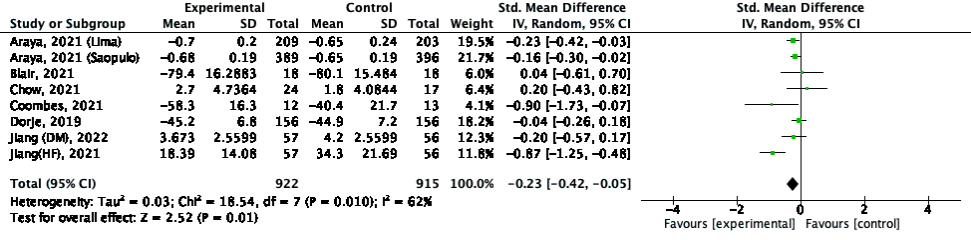
Quality of life for short term [26,27,32,34,37,39,44].

Seven studies were included. However, 1 study [27] considered each of the 2 real samples, so they were included in the meta-analysis separately. Therefore, a meta-analysis was performed as 8 studies [26,27,32,34,37,39,44]. There was a significant increase in improvement for the combination intervention in the short term (SMD –0.23; 95% CI –0.42 to –0.05; *P*<.001; *I*^2^=62%; Figure 5 [26,27,32,34,37,39,44]).

#### Medium Term

**Figure 6:**
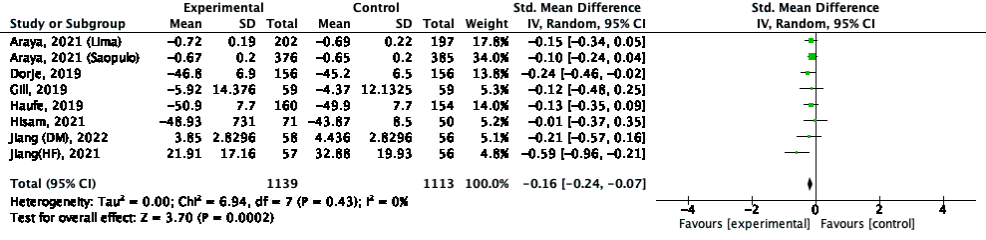
Quality of life for medium term [20,27,34,37,40,41,44].

Seven studies were included. However, 1 study [27] considered each of the 2 real samples, so they were included in the meta-analysis separately. Therefore, a meta-analysis was performed as 8 studies [20,27,34,37,40,41,44]. There was a significant increase in improvement for the combination intervention (SMD –0.16; 95% CI –0.24 to –0.07; *P*<.001; *I*^2^=0%; Figure 6 [20,27,34,37,40,41,44]).

#### Long Term

**Figure 7:**
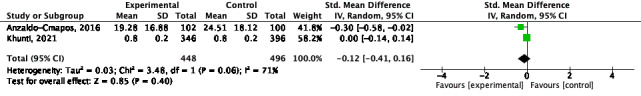
Quality of life for long term [25,30].

One study was excluded because data was not available [24]. A meta-analysis was performed on 2 studies [25,30]. There was no significant increase in improvement for the combination intervention (SMD –0.12; 95% CI –0.41 to 0.16; *P*=.04; *I*^2^=71%; Figure 7 [25,30]).

#### Physical Activity

We performed a meta-analysis of the outcome of physical activity, dividing the data into subjective (questionnaire) and objective (number of steps).

### Objective Outcomes of Physical Activity

#### Overview

It was not possible to perform a meta-analysis for the medium and long terms because there was less than 1 study that corresponded to this time.

#### Short Term

**Figure 8:**
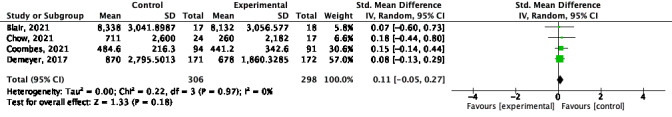
Physical activity (steps) for short term [26,28,32,39].

Meta-analysis was conducted in 4 studies [26,28,32,39]. There was no significant increase in improvement for the combination intervention (SMD 0.11; 95% CI –0.05 to 0.27; *P*=.18; *I*^2^=0%; Figure 8 [26,28,32,39]).

### Subjective Outcomes of Physical Activity

#### Short Term

**Figure 9:**
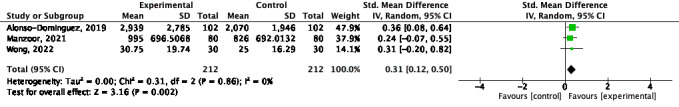
Physical activity (subjective) for short term [19,21,35].

A meta-analysis of 3 studies was conducted [19,21,35]. There was a significant increase in improvement for the combination intervention (SMD 0.31; 95% CI 0.12-0.50; *P*=.002; *I*^2^=0%; Figure 9 [19,21,35]).

#### Medium Term

**Figure 10:**
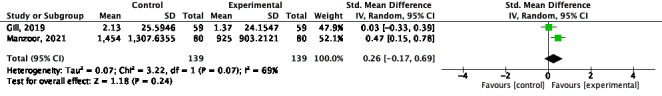
Physical activity (subjective) for medium term [19,40].

Meta-analysis was conducted in 2 studies [19,40]. There was no significant increase in improvement for the combination intervention (SMD 0.26; 95% CI –0.17 to 0.69; *P*=.24; *I*^2^=69%; Figure 10 [19,40]).

#### Long Term

**Figure 11:**
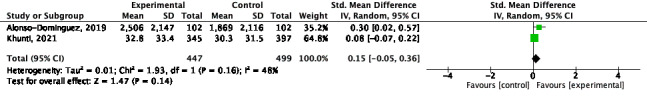
Physical activity (subjective) for long term [21,30].

Meta-analysis was conducted in 2 studies [21,30]. There was no significant increase in improvement for the combination intervention (SMD 0.15; 95% CI –0.05 to 0.36; *P*=.14; *I*^2^=48%; Figure 11 [21,30]).

## Discussion

### Principal Findings

This systematic review and meta-analysis showed that the combination of mHealth and health professional–led interventions improved health-related outcomes such as HbA_1c_ and QoL in chronic diseases. These effects were significant in the short and medium terms and there was no significant long term. For physical activity, there were no differences in intervention effects except for the short term as measured by questionnaires.

### HbA_1c_

In this systematic review, studies targeting patients with chronic diseases were included, and among the 26 studies, 12 incorporated HbA_1c_ as an outcome measure. In a meta-analysis that used mobile apps for lifestyle modification among diabetes patients, similar to our study, improvements in HbA_1c_ levels were demonstrated [45]. Furthermore, the meta-analysis indicated significant long-term effects in its subgroup analysis, defining long-term as 9-12 months [45], which differs from our study’s definition. The impact of mHealth was indicated to diminish in the long term due to factors such as adherence fatigue [46]. However, another meta-analysis found that digital health–led diabetes self-management education and support (including mHealth, eHealth, and interventions using social networking services) were effective in improving HbA_1c_ at 6 and 12 months [47]. The discrepancies between our study and the other meta-analysis may be due to some factors. These factors include variations in digital health interventions, professional involvement levels, patient populations, and adherence. The other meta-analysis also included mHealth, eHealth, and interventions using social networking services, which may offer different engagement and support levels. These differences in intervention design and implementation could contribute to the varying long-term effectiveness observed. An overview of the studies we included in our systematic review, for example, Anzaldo-Campos et al [25] demonstrated that a smartphone app used for diabetes management significantly improved HbA_1c_ levels over a 6-month period, highlighting the potential of app-based interventions for glycemic control. In contrast, Franc et al [22] found that a telemonitoring system improved HbA_1c_ levels significantly over 12 months, suggesting that the integration of professional support enhances the effectiveness of digital health interventions. The differing results could be due to the varying levels of professional involvement and continuous monitoring provided in the telemonitoring system, which may help sustain patient adherence and engagement over a longer period. Additionally, Jiang et al [37] found that a nurse-led smartphone intervention significantly reduced HbA_1c_ levels over 6 months in patients with type 2 diabetes, emphasizing the importance of professional guidance in mHealth interventions. Conversely, Zhang et al [36] reported that a self-guided mobile app intervention without professional support did not result in significant improvements in HbA_1c_ levels, highlighting the potential limitations of mHealth interventions lacking continuous professional involvement. Our findings suggest that while the combination of mHealth and health professional–led interventions can be as diverse as those described above, continuous feedback and support to patients through other digital interventions to enhance engagement may be a key factor in achieving long-term benefits [48].

### QoL

QoL was the most prevalent outcome measure among the studies included in this study. The results of our meta-analysis suggested that combining mHealth and health professional–led intervention can significantly improve QoL in chronic diseases in the short and medium terms. Studies conducting meta-analyses on the QoL in mHealth interventions for patients with chronic diseases are limited [49-51]. In a meta-analysis by Qin et al [50], mobile app–based interventions improved the QoL (SMD 0.39; 95% CI 0.27-0.51; *P*<.001) of patients with cancer. They emphasized that short-term interventions in particular (duration of 3 months or less), physician-patient interaction interventions, and cognitive behavioral therapy–based interventions may be most effective in improving QoL, which was consistent with this study. While extending beyond the scope of mHealth interventions, there is only 1 reported meta-analysis that indicated long-term improvements in QoL (mean difference 0.92; 95% CI 0.06-1.78; *P*=.04) resulting from cardiac telerehabilitation for patients with coronary artery disease [52]. For instance, Araya et al [27] found that a digital intervention combined with professional support significantly improved QoL in patients with cardiovascular diseases over 12 months. This improvement could be attributed to the continuous monitoring and personalized feedback provided by health care professionals, which likely enhanced patient adherence and engagement with the intervention. In contrast, Blasco et al [24] reported improvements in QoL among patients with acute coronary syndrome using a web-based telemonitoring system. The significant improvements observed in Blasco et al [24] study might be due to the comprehensive nature of the telemonitoring system, which included regular remote check-ins and the ability to promptly address patient concerns, thereby providing a sense of security and continuous care. Similarly, Manzoor et al [19] demonstrated that the mHealth-augmented cardiac rehabilitation program significantly improved QoL in patients with postacute coronary syndrome over 24 weeks, underscoring the importance of combining digital interventions with structured professional support. On the other hand, Wong et al [35] found that a self-guided mHealth intervention without professional involvement had limited effects on improving QoL, indicating that professional support is crucial for maximizing the benefits of digital health interventions. In our meta-analysis, focusing solely on mHealth interventions, it might be possible to identify long-term effects on the improvement of QoL by expanding the scope to include telehealth intervention or telerehabilitation as well.

### Physical Activity

The results of our meta-analysis suggested that physical activity had no significant effect except for the short-term effect of subjective assessment. One reason for this might be that many mHealth interventions did not include specifications or support to promote physical activity. This could be attributed to the predominant focus of many interventions on monitoring blood data and providing advice [22,24,25,29,31,33,34,36,42,43], potentially falling short of implementing behavior changes that could lead to promoting physical activity. In fact, among the 26 studies, only 8 studies adopted physical activity as the primary outcome. As an example of a study that adopted physical activity as the primary outcome, Gill et al [40] examined the effects of using a mobile app focused primarily on increasing physical activity and professional support for participants at risk for chronic disease and reported not only an increase in steps over the next 6 months but also maintenance of steps after 1 year. Dorje et al [44] found that a mHealth intervention including exercise modules improved physical activity levels in patients with coronary heart disease over 12 months, highlighting the potential of structured digital interventions. Conversely, Demeyer et al [28] reported that a self-guided digital intervention without professional support did not significantly increase physical activity levels in patients with chronic obstructive pulmonary disease, suggesting the need for continuous professional involvement to achieve sustained behavioral changes. In a different disease from the above, Blair et al [26] showed that a combination of mHealth and professional-led interventions for patients with cancer increased the number of steps taken, although there was no effect on outcomes related to sedentary behavior among physical activity measures. Thus, it would be valuable to identify in future studies which chronic diseases are more likely to improve physical activity with a combination of mHealth and professional-led interventions. Stavric et al [53] showed that digital physical activity and exercise interventions had positive effects on self-reported physical activity in people with chronic diseases, but not on objectively measured physical activity, which supports the results of our study. Their meta-analysis included self-guided digital mHealth interventions [53]. Therefore, there is room for future research to investigate whether self-directed digital or mHealth interventions or a combination of mHealth and health professional–led interventions, have more positive effects on physical activity.

### Implications for Practice

The results of our study suggest that combining mHealth with professionally led interventions can be effective in chronic disease management, particularly in short and medium terms. This combination approach shows potential for improving important health outcomes such as HbA_1c_ and QoL. However, the lack of long-term effects indicates the need for sustained and adaptive intervention strategies. Health care providers should consider integrating ongoing digital support and feedback mechanisms to maintain patient engagement and adherence over time. In addition, the involvement of health care professionals in mHealth interventions should be designed to provide personalized and digital support, leveraging technologies such as telemedicine and mobile apps. For example, health care organizations should invest in training and infrastructure to support the seamless integration of mHealth technologies into traditional care models. This can improve the scalability and effectiveness of chronic disease management programs, ultimately leading to better patient outcomes and reduced health care costs.

### Limitations

First, a major limitation of our study is the ambiguity in the definition of “health professional–led intervention.” Our research focused on a combination of such interventions with mHealth strategies. However, the term may be misleading, as many studies typically include some level of health professional involvement in routine care. In contrast, our study specifically included cases with a more active role for health professionals than is typically seen in routine care. Additionally, the definition of the control group varied across studies. Some studies included only general care, while others provided mHealth devices without additional interventions. Second, our focus on health-related outcomes in chronic diseases limited our ability to conduct comprehensive meta-analyses due to the diverse nature of these diseases and their outcomes. Finally, we did not assess the cost-effectiveness of integrating health professional–led interventions with mHealth strategies. While the involvement of health professionals could potentially improve intervention effectiveness and reduce dropout rates, it could also lead to higher costs. Although mHealth interventions are known to struggle with high dropout rates [54], the cost implications of incorporating health professional support need to be further explored in future studies.

### Conclusions

This systematic review and meta-analysis showed that the combined mHealth and health professional–led intervention had positive effects on HbA_1c_ and QoL in chronic conditions in short and medium terms. Positive effects on physical activity were only observed in the short term as measured by questionnaires. To achieve long-term effects of more than 1 year, it may be necessary to implement digital interventions that provide continuous feedback and support, tailored to a combination of mHealth and health professional–led interventions. Future research should explore the sustainability of these interventions over extended periods and investigate the specific components of mHealth and health professional input that contribute most to positive outcomes. Additionally, the integration of advanced technologies such as artificial intelligence and machine learning could further enhance the personalization and effectiveness of mHealth interventions.

## Appendix

Multimedia Appendix 1 PRISMA (Preferred Reporting Items for Systematic Reviews and Meta-Analyses) checklist.

Multimedia Appendix 2 Search strategy.

Multimedia Appendix 3 Characteristics of included studies with author names beginning with A to Z.

Multimedia Appendix 4 Risk of bias assessment.

Multimedia Appendix 5 Certainty of the evidence according to the GRADE (Grading of Recommendations Assessment, Development, and Evaluation) approach.

## Abbreviations

GRADE: Grading of Recommendations Assessment, Development, and Evaluation
mHealth: mobile health
PRISMA: Preferred Reporting Items for Systematic Reviews and Meta-analyses
QoL: quality of life
SMD: standardized mean difference
