# Supplementary material for: The Effect of Combining mHealth and Health Professional–Led Intervention for Improving Health-Related Outcomes in Chronic Diseases: Systematic Review and Meta-Analysis
Source: Interact J Med Res. 2025 Jan 20;14:e55835. doi: 10.2196/55835 (PMC11791457; doi:10.2196/55835)
Supplement: Multimedia Appendix 2 [file ijmr_v14i1e55835_app2.docx]

APPENDIX 1: Search Strategy

MEDLINE

| # |  |
| --- | --- |
| 1 | "Chronic Disease"[MH] OR (chronic*[TIAB] AND (disease*[TIAB] OR condition*[TIAB] OR ill[TIAB] OR illness*[TIAB])) |
| 2 | "Diabetes Mellitus"[MH] OR diabet*[TIAB] |
| 3 | ((kidney[TIAB] OR renal[TIAB]) AND (failure*[TIAB] OR disease*[TIAB])) |
| 4 | Heart Diseases[MH] OR cardi*[TIAB] OR heart*[TIAB] OR coronary*[TIAB] OR angina*[TIAB] OR myocard*[TIAB] OR ventricul*[TIAB] OR "atrioventricul*"[TIAB] OR pericard*[TIAB] OR "atrial*"[TIAB] OR endocardi*[TIAB] OR arrhythmi*[TIAB] OR thrombo*[TIAB] OR tachy*[TIAB] OR "brady*"[TIAB] OR fibrillat*[TIAB] |
| 5 | Stroke[MH] OR stroke[TIAB] OR Strokes[TIAB] OR Cerebral Vascular[TIAB] OR cerebrovascular[TIAB] |
| 6 | COPD[TIAB] OR ((obstruct*[TIAB]) AND (pulmonary[TIAB] OR lung*[TIAB] OR airway*[TIAB] OR airflow*[TIAB] OR bronch*[TIAB] OR respirat*[TIAB])) |
| 7 | obesity[MH] OR obes*[TIAB] OR overweight*[TIAB] OR "over weight*"[TIAB] OR Metabolic Syndrome[MH] |
| 8 | #1 OR #2 OR #3 OR #4 OR #5 OR #6 OR #7 |
|  | (((((("Chronic Disease"[MH] OR (chronic*[TIAB] AND (disease*[TIAB] OR condition*[TIAB] OR ill[TIAB] OR illness*[TIAB]))) OR ("Diabetes Mellitus"[MH] OR diabet*[TIAB])) OR (((kidney[TIAB] OR renal[TIAB]) AND (failure*[TIAB] OR disease*[TIAB])))) OR (Heart Diseases[MH] OR cardi*[TIAB] OR heart*[TIAB] OR coronary*[TIAB] OR angina*[TIAB] OR myocard*[TIAB] OR ventricul*[TIAB] OR "atrioventricul*"[TIAB] OR pericard*[TIAB] OR "atrial*"[TIAB] OR endocardi*[TIAB] OR arrhythmi*[TIAB] OR thrombo*[TIAB] OR tachy*[TIAB] OR "brady*"[TIAB] OR fibrillat*[TIAB])) OR (Stroke[MH] OR stroke[TIAB] OR Strokes[TIAB] OR Cerebral Vascular[TIAB] OR cerebrovascular[TIAB])) OR (COPD[TIAB] OR ((obstruct*[TIAB]) AND (pulmonary[TIAB] OR lung*[TIAB] OR airway*[TIAB] OR airflow*[TIAB] OR bronch*[TIAB] OR respirat*[TIAB])))) OR (obesity[MH] OR obes*[TIAB] OR overweight*[TIAB] OR "over weight*"[TIAB] OR Metabolic Syndrome[MH]) |
| 9 | mhealth[TIAB] OR m health[TIAB] OR eHealth[TIAB] OR e health[TIAB] OR digital health[TIAB] OR Telemedicine[MH] OR Mobile Applications[MH] OR mobile[TIAB] OR mobiles[TIAB] OR app[TIAB] OR apps[TIAB] OR Application[TIAB] OR Applications[TIAB] OR Cell Phone[MH] OR Cell Phone Use[MH] OR Computers, Handheld[MH] OR smartphone*[TIAB] OR smart phone*[TIAB] OR cellular phone*[TIAB] OR cell phone*[TIAB] OR Wearable Electronic Devices[MH] OR wearable[TIAB] OR smartwatch*[TIAB] OR smart watch*[TIAB] OR tablet*[TIAB] |
| 10 | Health Personnel[MH] OR (health[TIAB] OR healthcare[TIAB] OR medical[TIAB] OR clinical[TIAB]) AND (manager*[TIAB] OR personnel*[TIAB] OR staff[TIAB] OR worker*[TIAB] OR provider*[TIAB] ) OR professional*[TIAB] OR specialist*[TIAB] OR physician*[TIAB] OR doctor*[TIAB] OR practitioner*[TIAB] OR clinician*[TIAB] OR nurse*[TIAB] OR "nursing assistant*"[TIAB] OR midwife[TIAB] OR midwives[TIAB] OR pharmacist*[TIAB] OR physiotherapist*[TIAB] OR dentist*[TIAB] OR radiographer*[TIAB] OR Nutritionist*[TIAB] OR Counselors[MH] OR Mentors[MH] OR therapist*[TIAB] |
| 11 | Mentoring[MH] OR coach*[TIAB] OR mentor*[TIAB] OR Counseling[MH] OR counsel*[TIAB] OR patient education as topic[MH] OR Communication[MH] OR communication*[TIAB] OR telecoach*[TIAB] OR telementor*[TIAB] |
| 12 | #10 OR #11 |
|  | (Health Personnel[MH] OR (health[TIAB] OR healthcare[TIAB] OR medical[TIAB] OR clinical[TIAB]) AND (manager*[TIAB] OR personnel*[TIAB] OR staff[TIAB] OR worker*[TIAB] OR provider*[TIAB] ) OR professional*[TIAB] OR specialist*[TIAB] OR physician*[TIAB] OR doctor*[TIAB] OR practitioner*[TIAB] OR clinician*[TIAB] OR nurse*[TIAB] OR "nursing assistant*"[TIAB] OR midwife[TIAB] OR midwives[TIAB] OR pharmacist*[TIAB] OR physiotherapist*[TIAB] OR dentist*[TIAB] OR radiographer*[TIAB] OR Nutritionist*[TIAB] OR Counselors[MH] OR Mentors[MH] OR therapist*[TIAB]) OR (Mentoring[MH] OR coach*[TIAB] OR mentor*[TIAB] OR Counseling[MH] OR counsel*[TIAB] OR patient education as topic[MH] OR Communication[MH] OR communication*[TIAB] OR telecoach*[TIAB] OR telementor*[TIAB]) |
| 13 | #8 AND #9 AND #12 |
|  | (((((((("Chronic Disease"[MH] OR (chronic*[TIAB] AND (disease*[TIAB] OR condition*[TIAB] OR ill[TIAB] OR illness*[TIAB]))) OR ("Diabetes Mellitus"[MH] OR diabet*[TIAB])) OR (((kidney[TIAB] OR renal[TIAB]) AND (failure*[TIAB] OR disease*[TIAB])))) OR (Heart Diseases[MH] OR cardi*[TIAB] OR heart*[TIAB] OR coronary*[TIAB] OR angina*[TIAB] OR myocard*[TIAB] OR ventricul*[TIAB] OR "atrioventricul*"[TIAB] OR pericard*[TIAB] OR "atrial*"[TIAB] OR endocardi*[TIAB] OR arrhythmi*[TIAB] OR thrombo*[TIAB] OR tachy*[TIAB] OR "brady*"[TIAB] OR fibrillat*[TIAB])) OR (Stroke[MH] OR stroke[TIAB] OR Strokes[TIAB] OR Cerebral Vascular[TIAB] OR cerebrovascular[TIAB])) OR (COPD[TIAB] OR ((obstruct*[TIAB]) AND (pulmonary[TIAB] OR lung*[TIAB] OR airway*[TIAB] OR airflow*[TIAB] OR bronch*[TIAB] OR respirat*[TIAB])))) OR (obesity[MH] OR obes*[TIAB] OR overweight*[TIAB] OR "over weight*"[TIAB] OR Metabolic Syndrome[MH])) AND (mhealth[TIAB] OR m health[TIAB] OR eHealth[TIAB] OR e health[TIAB] OR digital health[TIAB] OR Telemedicine[MH] OR Mobile Applications[MH] OR mobile[TIAB] OR mobiles[TIAB] OR app[TIAB] OR apps[TIAB] OR Application[TIAB] OR Applications[TIAB] OR Cell Phone[MH] OR Cell Phone Use[MH] OR Computers, Handheld[MH] OR smartphone*[TIAB] OR smart phone*[TIAB] OR cellular phone*[TIAB] OR cell phone*[TIAB] OR Wearable Electronic Devices[MH] OR wearable[TIAB] OR smartwatch*[TIAB] OR smart watch*[TIAB] OR tablet*[TIAB])) AND ((Health Personnel[MH] OR (health[TIAB] OR healthcare[TIAB] OR medical[TIAB] OR clinical[TIAB]) AND (manager*[TIAB] OR personnel*[TIAB] OR staff[TIAB] OR worker*[TIAB] OR provider*[TIAB] ) OR professional*[TIAB] OR specialist*[TIAB] OR physician*[TIAB] OR doctor*[TIAB] OR practitioner*[TIAB] OR clinician*[TIAB] OR nurse*[TIAB] OR "nursing assistant*"[TIAB] OR midwife[TIAB] OR midwives[TIAB] OR pharmacist*[TIAB] OR physiotherapist*[TIAB] OR dentist*[TIAB] OR radiographer*[TIAB] OR Nutritionist*[TIAB] OR Counselors[MH] OR Mentors[MH] OR therapist*[TIAB]) OR (Mentoring[MH] OR coach*[TIAB] OR mentor*[TIAB] OR Counseling[MH] OR counsel*[TIAB] OR patient education as topic[MH] OR Communication[MH] OR communication*[TIAB] OR telecoach*[TIAB] OR telementor*[TIAB])) |
| 14 | #13 AND english[LA] |
|  | (((((((("Chronic Disease"[MH] OR (chronic*[TIAB] AND (disease*[TIAB] OR condition*[TIAB] OR ill[TIAB] OR illness*[TIAB]))) OR ("Diabetes Mellitus"[MH] OR diabet*[TIAB])) OR (((kidney[TIAB] OR renal[TIAB]) AND (failure*[TIAB] OR disease*[TIAB])))) OR (Heart Diseases[MH] OR cardi*[TIAB] OR heart*[TIAB] OR coronary*[TIAB] OR angina*[TIAB] OR myocard*[TIAB] OR ventricul*[TIAB] OR "atrioventricul*"[TIAB] OR pericard*[TIAB] OR "atrial*"[TIAB] OR endocardi*[TIAB] OR arrhythmi*[TIAB] OR thrombo*[TIAB] OR tachy*[TIAB] OR "brady*"[TIAB] OR fibrillat*[TIAB])) OR (Stroke[MH] OR stroke[TIAB] OR Strokes[TIAB] OR Cerebral Vascular[TIAB] OR cerebrovascular[TIAB])) OR (COPD[TIAB] OR ((obstruct*[TIAB]) AND (pulmonary[TIAB] OR lung*[TIAB] OR airway*[TIAB] OR airflow*[TIAB] OR bronch*[TIAB] OR respirat*[TIAB])))) OR (obesity[MH] OR obes*[TIAB] OR overweight*[TIAB] OR "over weight*"[TIAB] OR Metabolic Syndrome[MH])) AND (mhealth[TIAB] OR m health[TIAB] OR eHealth[TIAB] OR e health[TIAB] OR digital health[TIAB] OR Telemedicine[MH] OR Mobile Applications[MH] OR mobile[TIAB] OR mobiles[TIAB] OR app[TIAB] OR apps[TIAB] OR Application[TIAB] OR Applications[TIAB] OR Cell Phone[MH] OR Cell Phone Use[MH] OR Computers, Handheld[MH] OR smartphone*[TIAB] OR smart phone*[TIAB] OR cellular phone*[TIAB] OR cell phone*[TIAB] OR Wearable Electronic Devices[MH] OR wearable[TIAB] OR smartwatch*[TIAB] OR smart watch*[TIAB] OR tablet*[TIAB])) AND ((Health Personnel[MH] OR (health[TIAB] OR healthcare[TIAB] OR medical[TIAB] OR clinical[TIAB]) AND (manager*[TIAB] OR personnel*[TIAB] OR staff[TIAB] OR worker*[TIAB] OR provider*[TIAB] ) OR professional*[TIAB] OR specialist*[TIAB] OR physician*[TIAB] OR doctor*[TIAB] OR practitioner*[TIAB] OR clinician*[TIAB] OR nurse*[TIAB] OR "nursing assistant*"[TIAB] OR midwife[TIAB] OR midwives[TIAB] OR pharmacist*[TIAB] OR physiotherapist*[TIAB] OR dentist*[TIAB] OR radiographer*[TIAB] OR Nutritionist*[TIAB] OR Counselors[MH] OR Mentors[MH] OR therapist*[TIAB]) OR (Mentoring[MH] OR coach*[TIAB] OR mentor*[TIAB] OR Counseling[MH] OR counsel*[TIAB] OR patient education as topic[MH] OR Communication[MH] OR communication*[TIAB] OR telecoach*[TIAB] OR telementor*[TIAB])) AND english[LA] |
| 15 | (randomized controlled trial[pt] OR controlled clinical trial[pt] OR randomized[tiab] OR placebo[tiab] OR drug therapy[sh] OR randomly[tiab] OR trial[tiab] OR groups[tiab] NOT (animals [mh] NOT humans [mh])) |
| 16 | (randomized controlled trial[pt] OR controlled clinical trial[pt] OR randomized[tiab] OR placebo[tiab] OR clinical trials as topic[mesh:noexp] OR randomly[tiab] OR trial[ti] NOT (animals[mh] NOT humans [mh])) |
| 17 | #14 AND #15 |
|  | ((((((((("Chronic Disease"[MH] OR (chronic*[TIAB] AND (disease*[TIAB] OR condition*[TIAB] OR ill[TIAB] OR illness*[TIAB]))) OR ("Diabetes Mellitus"[MH] OR diabet*[TIAB])) OR (((kidney[TIAB] OR renal[TIAB]) AND (failure*[TIAB] OR disease*[TIAB])))) OR (Heart Diseases[MH] OR cardi*[TIAB] OR heart*[TIAB] OR coronary*[TIAB] OR angina*[TIAB] OR myocard*[TIAB] OR ventricul*[TIAB] OR "atrioventricul*"[TIAB] OR pericard*[TIAB] OR "atrial*"[TIAB] OR endocardi*[TIAB] OR arrhythmi*[TIAB] OR thrombo*[TIAB] OR tachy*[TIAB] OR "brady*"[TIAB] OR fibrillat*[TIAB])) OR (Stroke[MH] OR stroke[TIAB] OR Strokes[TIAB] OR Cerebral Vascular[TIAB] OR cerebrovascular[TIAB])) OR (COPD[TIAB] OR ((obstruct*[TIAB]) AND (pulmonary[TIAB] OR lung*[TIAB] OR airway*[TIAB] OR airflow*[TIAB] OR bronch*[TIAB] OR respirat*[TIAB])))) OR (obesity[MH] OR obes*[TIAB] OR overweight*[TIAB] OR "over weight*"[TIAB] OR Metabolic Syndrome[MH])) AND (mhealth[TIAB] OR m health[TIAB] OR eHealth[TIAB] OR e health[TIAB] OR digital health[TIAB] OR Telemedicine[MH] OR Mobile Applications[MH] OR mobile[TIAB] OR mobiles[TIAB] OR app[TIAB] OR apps[TIAB] OR Application[TIAB] OR Applications[TIAB] OR Cell Phone[MH] OR Cell Phone Use[MH] OR Computers, Handheld[MH] OR smartphone*[TIAB] OR smart phone*[TIAB] OR cellular phone*[TIAB] OR cell phone*[TIAB] OR Wearable Electronic Devices[MH] OR wearable[TIAB] OR smartwatch*[TIAB] OR smart watch*[TIAB] OR tablet*[TIAB])) AND ((Health Personnel[MH] OR (health[TIAB] OR healthcare[TIAB] OR medical[TIAB] OR clinical[TIAB]) AND (manager*[TIAB] OR personnel*[TIAB] OR staff[TIAB] OR worker*[TIAB] OR provider*[TIAB] ) OR professional*[TIAB] OR specialist*[TIAB] OR physician*[TIAB] OR doctor*[TIAB] OR practitioner*[TIAB] OR clinician*[TIAB] OR nurse*[TIAB] OR "nursing assistant*"[TIAB] OR midwife[TIAB] OR midwives[TIAB] OR pharmacist*[TIAB] OR physiotherapist*[TIAB] OR dentist*[TIAB] OR radiographer*[TIAB] OR Nutritionist*[TIAB] OR Counselors[MH] OR Mentors[MH] OR therapist*[TIAB]) OR (Mentoring[MH] OR coach*[TIAB] OR mentor*[TIAB] OR Counseling[MH] OR counsel*[TIAB] OR patient education as topic[MH] OR Communication[MH] OR communication*[TIAB] OR telecoach*[TIAB] OR telementor*[TIAB])) AND english[LA]) AND ((randomized controlled trial[pt] OR controlled clinical trial[pt] OR randomized[tiab] OR placebo[tiab] OR drug therapy[sh] OR randomly[tiab] OR trial[tiab] OR groups[tiab] NOT (animals [mh] NOT humans [mh]))) |
| 18 | #14 AND #16 |
|  | ((((((((("Chronic Disease"[MH] OR (chronic*[TIAB] AND (disease*[TIAB] OR condition*[TIAB] OR ill[TIAB] OR illness*[TIAB]))) OR ("Diabetes Mellitus"[MH] OR diabet*[TIAB])) OR (((kidney[TIAB] OR renal[TIAB]) AND (failure*[TIAB] OR disease*[TIAB])))) OR (Heart Diseases[MH] OR cardi*[TIAB] OR heart*[TIAB] OR coronary*[TIAB] OR angina*[TIAB] OR myocard*[TIAB] OR ventricul*[TIAB] OR "atrioventricul*"[TIAB] OR pericard*[TIAB] OR "atrial*"[TIAB] OR endocardi*[TIAB] OR arrhythmi*[TIAB] OR thrombo*[TIAB] OR tachy*[TIAB] OR "brady*"[TIAB] OR fibrillat*[TIAB])) OR (Stroke[MH] OR stroke[TIAB] OR Strokes[TIAB] OR Cerebral Vascular[TIAB] OR cerebrovascular[TIAB])) OR (COPD[TIAB] OR ((obstruct*[TIAB]) AND (pulmonary[TIAB] OR lung*[TIAB] OR airway*[TIAB] OR airflow*[TIAB] OR bronch*[TIAB] OR respirat*[TIAB])))) OR (obesity[MH] OR obes*[TIAB] OR overweight*[TIAB] OR "over weight*"[TIAB] OR Metabolic Syndrome[MH])) AND (mhealth[TIAB] OR m health[TIAB] OR eHealth[TIAB] OR e health[TIAB] OR digital health[TIAB] OR Telemedicine[MH] OR Mobile Applications[MH] OR mobile[TIAB] OR mobiles[TIAB] OR app[TIAB] OR apps[TIAB] OR Application[TIAB] OR Applications[TIAB] OR Cell Phone[MH] OR Cell Phone Use[MH] OR Computers, Handheld[MH] OR smartphone*[TIAB] OR smart phone*[TIAB] OR cellular phone*[TIAB] OR cell phone*[TIAB] OR Wearable Electronic Devices[MH] OR wearable[TIAB] OR smartwatch*[TIAB] OR smart watch*[TIAB] OR tablet*[TIAB])) AND ((Health Personnel[MH] OR (health[TIAB] OR healthcare[TIAB] OR medical[TIAB] OR clinical[TIAB]) AND (manager*[TIAB] OR personnel*[TIAB] OR staff[TIAB] OR worker*[TIAB] OR provider*[TIAB] ) OR professional*[TIAB] OR specialist*[TIAB] OR physician*[TIAB] OR doctor*[TIAB] OR practitioner*[TIAB] OR clinician*[TIAB] OR nurse*[TIAB] OR "nursing assistant*"[TIAB] OR midwife[TIAB] OR midwives[TIAB] OR pharmacist*[TIAB] OR physiotherapist*[TIAB] OR dentist*[TIAB] OR radiographer*[TIAB] OR Nutritionist*[TIAB] OR Counselors[MH] OR Mentors[MH] OR therapist*[TIAB]) OR (Mentoring[MH] OR coach*[TIAB] OR mentor*[TIAB] OR Counseling[MH] OR counsel*[TIAB] OR patient education as topic[MH] OR Communication[MH] OR communication*[TIAB] OR telecoach*[TIAB] OR telementor*[TIAB])) AND english[LA]) AND ((randomized controlled trial[pt] OR controlled clinical trial[pt] OR randomized[tiab] OR placebo[tiab] OR clinical trials as topic[mesh:noexp] OR randomly[tiab] OR trial[ti] NOT (animals[mh] NOT humans [mh]))) |
| 19 | "Randomized Controlled Trial"[pt] OR "Controlled Clinical Trial"[pt] OR "Pragmatic Clinical Trial"[pt] OR "Equivalence Trial"[pt] OR "Clinical Trial, Phase III"[pt] OR "Randomized Controlled Trials as Topic"[mh] OR "Controlled Clinical Trials as Topic"[mh] OR "Random Allocation"[mh] OR "Double-Blind Method"[mh] OR "Single-Blind Method"[mh] OR Placebos[Mesh:NoExp] OR "Control Groups"[mh] OR (random*[tiab] OR sham[tiab] OR placebo*[tiab]) OR ((singl*[tiab] OR doubl*[tiab]) AND (blind*[tiab] OR dumm*[tiab] OR mask*[tiab])) OR ((tripl*[tiab] OR trebl*[tiab]) AND (blind*[tiab] OR dumm*[tiab] OR mask*[tiab])) OR (control*[tiab] AND (study[tiab] OR studies[tiab] OR trial*[tiab] OR group*[tiab])) OR (Nonrandom*[tiab] OR "non random*"[tiab] OR "non-random*"[tiab] OR "quasi-random*"[tiab] OR quasirandom*[tiab]) OR allocated[tiab] OR (("open label"[tiab] OR "open-label"[tiab]) AND (study[tiab] OR studies[tiab] OR trial*[tiab])) OR ((equivalence[tiab] OR superiority[tiab] OR "non-inferiority"[tiab] OR noninferiority[tiab]) AND (study[tiab] OR studies[tiab] OR trial*[tiab])) OR ("pragmatic study"[tiab] OR "pragmatic studies"[tiab]) OR ((pragmatic[tiab] OR practical[tiab]) AND trial*[tiab]) OR ((quasiexperimental[tiab] OR "quasi-experimental"[tiab]) AND (study[tiab] OR studies[tiab] OR trial*[tiab])) OR (phase[ti] AND (III[ti] OR 3[ti]) AND (study[ti] OR studies[ti] OR trial*[ti])) OR (phase[ot] AND (III[ot] OR 3[ot]) AND (study[ot] OR studies[ot] OR trial*[ot])) |
|  | #14 AND #19 |

CENTRAL

| # | 検索式 |
| --- | --- |
| 1 | MeSH descriptor: [Chronic Disease] explode all trees OR( (chronic*):ti,ab,kw AND ((disease*):ti,ab,kw OR (condition*):ti,ab,kw OR (ill):ti,ab,kw OR (illness*):ti,ab,kw)) |
| 2 | MeSH descriptor: [Diabetes Mellitus] explode all trees OR (diabet*):ti,ab,kw |
| 3 | ((kidney):ti,ab,kw OR (renal):ti,ab,kw) AND ((failure*):ti,ab,kw OR (disease*):ti,ab,kw) |
| 4 | MeSH descriptor: [Heart Diseases] explode all trees OR (cardi*):ti,ab,kw OR (heart*):ti,ab,kw OR (coronary*):ti,ab,kw OR (angina*):ti,ab,kw OR (myocard*):ti,ab,kw OR (ventricul*):ti,ab,kw OR (atrioventricul*):ti,ab,kw OR (pericard*):ti,ab,kw OR (atrial*):ti,ab,kw OR (endocardi*):ti,ab,kw OR (arrhythmi*):ti,ab,kw OR (thrombo*):ti,ab,kw OR (tachy*):ti,ab,kw OR (brady*):ti,ab,kw OR (fibrillat*):ti,ab,kw |
| 5 | MeSH descriptor: [Stroke] explode all trees OR (stroke):ti,ab,kw OR (Strokes):ti,ab,kw OR (Cerebral Vascular):ti,ab,kw OR (cerebrovascular):ti,ab,kw |
| 6 | ((COPD):ti,ab,kw OR (((obstruct*):ti,ab,kw) AND ((pulmonary):ti,ab,kw OR (lung*):ti,ab,kw OR (airway*):ti,ab,kw OR (airflow*):ti,ab,kw OR (bronch*):ti,ab,kw OR (respirat*):ti,ab,kw))) |
| 7 | MeSH descriptor: [obesity] explode all trees OR (obes*):ti,ab,kw OR (overweight*):ti,ab,kw OR (over NEXT weight*):ti,ab,kw OR MeSH descriptor: [Metabolic Syndrome] explode all trees |
| 8 | #1 OR #2 OR #3 OR #4 OR #5 OR #6 OR #7 |
| 9 | (mhealth):ti,ab,kw OR ("m health"):ti,ab,kw OR (m-health):ti,ab,kw OR (eHealth):ti,ab,kw OR ("e health"):ti,ab,kw OR (e-health):ti,ab,kw OR ("digital health"):ti,ab,kw OR MeSH descriptor: [Telemedicine] explode all trees OR MeSH descriptor: [Mobile Applications] explode all trees OR (mobile):ti,ab,kw OR (mobiles):ti,ab,kw OR (app):ti,ab,kw OR (apps):ti,ab,kw OR (Application):ti,ab,kw OR (Applications):ti,ab,kw OR MeSH descriptor: [Cell Phone] explode all trees OR MeSH descriptor: [Cell Phone Use] explode all trees OR MeSH descriptor: [Computers, Handheld] explode all trees OR (smartphone*):ti,ab,kw OR (smart NEXT phone*):ti,ab,kw OR (tablet*):ti,ab,kw OR (cellular NEXT phone*):ti,ab,kw OR (cell NEXT phone*):ti,ab,kw OR MeSH descriptor: [Wearable Electronic Devices] explode all trees OR (wearable):ti,ab,kw OR (smartwatch*):ti,ab,kw OR (smart NEXT watch*):ti,ab,kw |
| 10 | MeSH descriptor: [Health Personnel] explode all trees OR ((health):ti,ab,kw OR (healthcare):ti,ab,kw OR (medical):ti,ab,kw OR (clinical):ti,ab,kw) AND ((manager*):ti,ab,kw OR (personnel*):ti,ab,kw OR (staff):ti,ab,kw OR (worker*):ti,ab,kw OR (provider*):ti,ab,kw ) OR (professional*):ti,ab,kw OR (specialist*):ti,ab,kw OR (physician*):ti,ab,kw OR (doctor*):ti,ab,kw OR (practitioner*):ti,ab,kw OR (clinician*):ti,ab,kw OR (nurse*):ti,ab,kw OR (nursing NEXT assistant*):ti,ab,kw OR (midwife):ti,ab,kw OR (midwives):ti,ab,kw OR (pharmacist*):ti,ab,kw OR (physiotherapist*):ti,ab,kw OR (dentist*):ti,ab,kw OR (radiographer*):ti,ab,kw OR (Nutritionist*):ti,ab,kw OR MeSH descriptor: [Counselors] explode all trees OR MeSH descriptor: [Mentors] explode all trees OR (therapist*):ti,ab,kw |
| 11 | MeSH descriptor: [Mentoring] explode all trees OR (coach*):ti,ab,kw OR (mentor*):ti,ab,kw OR MeSH descriptor: [Counseling] explode all trees OR (counsel*):ti,ab,kw OR MeSH descriptor: [patient education as topic] explode all trees OR MeSH descriptor: [Communication] explode all trees OR (communication):ti,ab,kw OR (telecoach*):ti,ab,kw OR (telementor*):ti,ab,kw |
| 12 | #10 OR #11 |
| 13 | #8 AND #9 AND #12 |

CINAHL

| # | 検索式 |
| --- | --- |
| 1 | MH "Chronic Disease+" OR (TI chronic* AND (TI disease* OR TI condition* OR TI "ill" OR TI illness*)) OR (AB chronic* AND (AB disease* OR AB condition* OR AB "ill" OR AB illness*)) |
| 2 | MH "Diabetes Mellitus+" OR TI diabet* OR AB diabet* |
| 3 | MH "Renal Insufficiency, Chronic+" OR ((TI "kidney" OR TI "renal") AND (TI failure* OR TI disease*)) OR ((AB "kidney" OR AB "renal") AND (AB failure* OR AB disease*)) |
| 4 | MH "Heart Diseases+" OR TI cardi* OR AB cardi* OR TI heart* OR AB heart* OR TI coronary* OR AB coronary* OR TI angina* OR AB angina* OR TI myocard* OR AB myocard* OR TI ventricul* OR AB ventricul* OR TI atrioventricul* OR AB atrioventricul* OR TI pericard* OR AB pericard* OR TI atrial* OR AB atrial* OR TI endocardi* OR AB endocardi* OR TI arrhythmi* OR AB arrhythmi* OR TI thrombo* OR AB thrombo* OR TI tachy* OR AB tachy* OR TI brady* OR AB brady* OR TI fibrillat* OR AB fibrillat* |
| 5 | MH "Stroke+" OR TI "stroke" OR AB "stroke" OR TI "strokes" OR AB "strokes" OR TI "cerebral vascular" OR AB "cerebral vascular" OR TI "cerebrovascular" OR AB "cerebrovascular" |
| 6 | MH "Pulmonary Disease, Chronic Obstructive+" OR TI "COPD" OR AB "COPD" OR (TI obstruct* AND (TI "pulmonary" OR TI lung* OR TI airway* OR TI airflow* OR TI bronch* OR TI respirat*)) OR (AB obstruct* AND (AB "pulmonary" OR AB lung* OR AB airway* OR AB airflow* OR AB bronch* OR AB respirat*)) |
| 7 | MH "Obesity+" OR TI obes* OR AB obes* OR TI overweight* OR AB overweight* OR TI "over weight*" OR AB "over weight*" OR MH "Metabolic Syndrome X+" |
| 8 | #1 OR #2 OR #3 OR #4 OR #5 OR #6 OR #7 |
|  | ( MH "Chronic Disease+" OR (TI chronic* AND (TI disease* OR TI condition* OR TI "ill" OR TI illness*)) OR (AB chronic* AND (AB disease* OR AB condition* OR AB "ill" OR AB illness*)) OR MH "Diabetes Mellitus+" OR TI diabet* OR AB diabet* OR MH "Renal Insufficiency, Chronic+" OR ((TI "kidney" OR TI "renal") AND (TI failure* OR TI disease*)) OR ((AB "kidney" OR AB "renal") AND (AB failure* OR AB disease*)) OR MH "Heart Diseases+" OR TI cardi* OR AB cardi* OR TI heart* OR AB heart* OR TI coronary* OR AB coronary* OR TI angina* OR AB angina* OR TI myocard* OR AB myocard* OR TI ventricul* OR AB ventricul* OR TI atrioventricul* OR AB atrioventricul* OR TI pericard* OR AB pericard* OR TI atrial* OR AB atrial* OR TI endocardi* OR AB endocardi* OR TI arrhythmi* OR AB arrhythmi* OR TI thrombo* OR AB thrombo* OR TI tachy* OR AB tachy* OR TI brady* OR AB brady* OR TI fibrillat* OR AB fibrillat* OR MH "Stroke+" OR TI "stroke" OR AB "stroke" OR TI "strokes" OR AB "strokes" OR TI "cerebral vascular" OR AB "cerebral vascular" OR TI "cerebrovascular" OR AB "cerebrovascular" OR MH "Pulmonary Disease, Chronic Obstructive+" OR TI "COPD" OR AB "COPD" OR (TI obstruct* AND (TI "pulmonary" OR TI lung* OR TI airway* OR TI airflow* OR TI bronch* OR TI respirat*)) OR (AB obstruct* AND (AB "pulmonary" OR AB lung* OR AB airway* OR AB airflow* OR AB bronch* OR AB respirat*)) OR MH "Obesity+" OR TI obes* OR AB obes* OR TI overweight* OR AB overweight* OR TI "over weight*" OR AB "over weight*" OR MH "Metabolic Syndrome X+" ) |
| 9 | ( TI "mhealth" OR AB "mhealth" OR TI "m health" OR AB "m health" OR TI "eHealth" OR AB "eHealth" OR TI "e health" OR AB "e health" OR TI "digital health" OR AB "digital health" OR MH "Digital Health+" OR MH "Telehealth+" OR MH "Mobile Applications" OR TI "mobile" OR AB "mobile" OR TI "mobiles" OR AB "mobiles" OR TI "app" OR AB "app" OR TI "apps" OR AB "apps" OR TI "Application" OR AB "Application" OR TI "Applications" OR AB "Applications" OR MH "Cellular Phone+" OR MH "Computers, Hand-Held+" OR TI smartphone* OR AB smartphone* OR TI "smart phone*" OR AB "smart phone*" OR TI "cellular phone*" OR AB "cellular phone*" OR TI "cell phone*" OR AB "cell phone*" OR TI "wearable" OR AB "wearable" OR TI smartwatch* OR AB smartwatch* OR TI "smart watch*" OR AB "smart watch*" OR TI tablet* OR AB tablet* ) |
| 10 | ( MH "Health Personnel+" OR ((TI "health" OR TI "healthcare" OR TI "medical" OR TI "clinical") AND (TI manager* OR TI personnel* OR TI "staff" OR TI worker* OR TI provider*)) OR ((AB "health" OR AB "healthcare" OR AB "medical" OR AB "clinical") AND (AB manager* OR AB personnel* OR AB "staff" OR AB worker* OR AB provider*)) OR TI professional* OR AB professional* OR TI specialist* OR AB specialist* OR TI physician* OR AB physician* OR TI doctor* OR AB doctor* OR TI practitioner* OR AB practitioner* OR TI clinician* OR AB clinician* OR TI nurse* OR AB nurse* OR TI "nursing assistant*" OR AB "nursing assistant*" OR TI "midwife" OR AB "midwife" OR TI "midwives" OR AB "midwives" OR TI pharmacist* OR AB pharmacist* OR TI therapist* OR AB therapist* OR TI physiotherapist* OR AB physiotherapist* OR TI dentist* OR AB dentist* OR TI radiographer* OR AB radiographer* OR TI nutritionist* OR AB nutritionist* OR MH "Counselors+" OR TI mentor* OR AB mentor* ) |
| 11 | ( MH "Mentorship" OR TI coach* OR AB coach* OR MH "Counseling+" OR TI counsel* OR AB counsel* OR MH "Patient Education+" OR MH "Communication+" OR TI communication* OR AB communication* OR TI telecoach* OR AB telecoach* OR TI telementor* OR AB telementor* ) |
| 12 | #10 OR #11 |
|  | ( MH "Health Personnel+" OR ((TI "health" OR TI "healthcare" OR TI "medical" OR TI "clinical") AND (TI manager* OR TI personnel* OR TI "staff" OR TI worker* OR TI provider*)) OR ((AB "health" OR AB "healthcare" OR AB "medical" OR AB "clinical") AND (AB manager* OR AB personnel* OR AB "staff" OR AB worker* OR AB provider*)) OR TI professional* OR AB professional* OR TI specialist* OR AB specialist* OR TI physician* OR AB physician* OR TI doctor* OR AB doctor* OR TI practitioner* OR AB practitioner* OR TI clinician* OR AB clinician* OR TI nurse* OR AB nurse* OR TI "nursing assistant*" OR AB "nursing assistant*" OR TI "midwife" OR AB "midwife" OR TI "midwives" OR AB "midwives" OR TI pharmacist* OR AB pharmacist* OR TI therapist* OR AB therapist* OR TI physiotherapist* OR AB physiotherapist* OR TI dentist* OR AB dentist* OR TI radiographer* OR AB radiographer* OR TI nutritionist* OR AB nutritionist* OR MH "Counselors+" OR TI mentor* OR AB mentor* ) OR ( MH "Mentorship" OR TI coach* OR AB coach* OR MH "Counseling+" OR TI counsel* OR AB counsel* OR MH "Patient Education+" OR MH "Communication+" OR TI communication* OR AB communication* OR TI telecoach* OR AB telecoach* OR TI telementor* OR AB telementor* ) |
| 13 | #8 AND #9 AND #12 |
|  | ( MH "Chronic Disease+" OR (TI chronic* AND (TI disease* OR TI condition* OR TI "ill" OR TI illness*)) OR (AB chronic* AND (AB disease* OR AB condition* OR AB "ill" OR AB illness*)) OR MH "Diabetes Mellitus+" OR TI diabet* OR AB diabet* OR MH "Renal Insufficiency, Chronic+" OR ((TI "kidney" OR TI "renal") AND (TI failure* OR TI disease*)) OR ((AB "kidney" OR AB "renal") AND (AB failure* OR AB disease*)) OR MH "Heart Diseases+" OR TI cardi* OR AB cardi* OR TI heart* OR AB heart* OR TI coronary* OR AB coronary* OR TI angina* OR AB angina* OR TI myocard* OR AB myocard* OR TI ventricul* OR AB ventricul* OR TI atrioventricul* OR AB atrioventricul* OR TI pericard* OR AB pericard* OR TI atrial* OR AB atrial* OR TI endocardi* OR AB endocardi* OR TI arrhythmi* OR AB arrhythmi* OR TI thrombo* OR AB thrombo* OR TI tachy* OR AB tachy* OR TI brady* OR AB brady* OR TI fibrillat* OR AB fibrillat* OR MH "Stroke+" OR TI "stroke" OR AB "stroke" OR TI "strokes" OR AB "strokes" OR TI "cerebral vascular" OR AB "cerebral vascular" OR TI "cerebrovascular" OR AB "cerebrovascular" OR MH "Pulmonary Disease, Chronic Obstructive+" OR TI "COPD" OR AB "COPD" OR (TI obstruct* AND (TI "pulmonary" OR TI lung* OR TI airway* OR TI airflow* OR TI bronch* OR TI respirat*)) OR (AB obstruct* AND (AB "pulmonary" OR AB lung* OR AB airway* OR AB airflow* OR AB bronch* OR AB respirat*)) OR MH "Obesity+" OR TI obes* OR AB obes* OR TI overweight* OR AB overweight* OR TI "over weight*" OR AB "over weight*" OR MH "Metabolic Syndrome X+" ) AND ( TI "mhealth" OR AB "mhealth" OR TI "m health" OR AB "m health" OR TI "eHealth" OR AB "eHealth" OR TI "e health" OR AB "e health" OR TI "digital health" OR AB "digital health" OR MH "Digital Health+" OR MH "Telehealth+" OR MH "Mobile Applications" OR TI "mobile" OR AB "mobile" OR TI "mobiles" OR AB "mobiles" OR TI "app" OR AB "app" OR TI "apps" OR AB "apps" OR TI "Application" OR AB "Application" OR TI "Applications" OR AB "Applications" OR MH "Cellular Phone+" OR MH "Computers, Hand-Held+" OR TI smartphone* OR AB smartphone* OR TI "smart phone*" OR AB "smart phone*" OR TI "cellular phone*" OR AB "cellular phone*" OR TI "cell phone*" OR AB "cell phone*" OR TI "wearable" OR AB "wearable" OR TI smartwatch* OR AB smartwatch* OR TI "smart watch*" OR AB "smart watch*" OR TI tablet* OR AB tablet* ) AND (( MH "Health Personnel+" OR ((TI "health" OR TI "healthcare" OR TI "medical" OR TI "clinical") AND (TI manager* OR TI personnel* OR TI "staff" OR TI worker* OR TI provider*)) OR ((AB "health" OR AB "healthcare" OR AB "medical" OR AB "clinical") AND (AB manager* OR AB personnel* OR AB "staff" OR AB worker* OR AB provider*)) OR TI professional* OR AB professional* OR TI specialist* OR AB specialist* OR TI physician* OR AB physician* OR TI doctor* OR AB doctor* OR TI practitioner* OR AB practitioner* OR TI clinician* OR AB clinician* OR TI nurse* OR AB nurse* OR TI "nursing assistant*" OR AB "nursing assistant*" OR TI "midwife" OR AB "midwife" OR TI "midwives" OR AB "midwives" OR TI pharmacist* OR AB pharmacist* OR TI therapist* OR AB therapist* OR TI physiotherapist* OR AB physiotherapist* OR TI dentist* OR AB dentist* OR TI radiographer* OR AB radiographer* OR TI nutritionist* OR AB nutritionist* OR MH "Counselors+" OR TI mentor* OR AB mentor* ) OR ( MH "Mentorship" OR TI coach* OR AB coach* OR MH "Counseling+" OR TI counsel* OR AB counsel* OR MH "Patient Education+" OR MH "Communication+" OR TI communication* OR AB communication* OR TI telecoach* OR AB telecoach* OR TI telementor* OR AB telementor* )) |
| 14 | ( MH ( randomized controlled trials OR double‐blind studies OR single‐blind studies OR random assignment OR pretest‐posttest design OR cluster sample ) OR TI ( randomised OR randomized ) OR AB random* OR TI trial OR ( (MH (sample size) AND AB (assigned OR allocated OR control)) ) OR MH ( placebos OR crossover design OR comparative studies ) OR AB ( (control W5 group) OR (cluster W3 RCT) OR PT (randomized controlled trial)) ) NOT ( ( MH animals+ OR MH (animal studies) OR TI (animal model*) ) NOT MH (human) ) |
| 15 | #13 AND #14 |
|  | ( MH "Chronic Disease+" OR (TI chronic* AND (TI disease* OR TI condition* OR TI "ill" OR TI illness*)) OR (AB chronic* AND (AB disease* OR AB condition* OR AB "ill" OR AB illness*)) OR MH "Diabetes Mellitus+" OR TI diabet* OR AB diabet* OR MH "Renal Insufficiency, Chronic+" OR ((TI "kidney" OR TI "renal") AND (TI failure* OR TI disease*)) OR ((AB "kidney" OR AB "renal") AND (AB failure* OR AB disease*)) OR MH "Heart Diseases+" OR TI cardi* OR AB cardi* OR TI heart* OR AB heart* OR TI coronary* OR AB coronary* OR TI angina* OR AB angina* OR TI myocard* OR AB myocard* OR TI ventricul* OR AB ventricul* OR TI atrioventricul* OR AB atrioventricul* OR TI pericard* OR AB pericard* OR TI atrial* OR AB atrial* OR TI endocardi* OR AB endocardi* OR TI arrhythmi* OR AB arrhythmi* OR TI thrombo* OR AB thrombo* OR TI tachy* OR AB tachy* OR TI brady* OR AB brady* OR TI fibrillat* OR AB fibrillat* OR MH "Stroke+" OR TI "stroke" OR AB "stroke" OR TI "strokes" OR AB "strokes" OR TI "cerebral vascular" OR AB "cerebral vascular" OR TI "cerebrovascular" OR AB "cerebrovascular" OR MH "Pulmonary Disease, Chronic Obstructive+" OR TI "COPD" OR AB "COPD" OR (TI obstruct* AND (TI "pulmonary" OR TI lung* OR TI airway* OR TI airflow* OR TI bronch* OR TI respirat*)) OR (AB obstruct* AND (AB "pulmonary" OR AB lung* OR AB airway* OR AB airflow* OR AB bronch* OR AB respirat*)) OR MH "Obesity+" OR TI obes* OR AB obes* OR TI overweight* OR AB overweight* OR TI "over weight*" OR AB "over weight*" OR MH "Metabolic Syndrome X+" ) AND ( TI "mhealth" OR AB "mhealth" OR TI "m health" OR AB "m health" OR TI "eHealth" OR AB "eHealth" OR TI "e health" OR AB "e health" OR TI "digital health" OR AB "digital health" OR MH "Digital Health+" OR MH "Telehealth+" OR MH "Mobile Applications" OR TI "mobile" OR AB "mobile" OR TI "mobiles" OR AB "mobiles" OR TI "app" OR AB "app" OR TI "apps" OR AB "apps" OR TI "Application" OR AB "Application" OR TI "Applications" OR AB "Applications" OR MH "Cellular Phone+" OR MH "Computers, Hand-Held+" OR TI smartphone* OR AB smartphone* OR TI "smart phone*" OR AB "smart phone*" OR TI "cellular phone*" OR AB "cellular phone*" OR TI "cell phone*" OR AB "cell phone*" OR TI "wearable" OR AB "wearable" OR TI smartwatch* OR AB smartwatch* OR TI "smart watch*" OR AB "smart watch*" OR TI tablet* OR AB tablet* ) AND (( MH "Health Personnel+" OR ((TI "health" OR TI "healthcare" OR TI "medical" OR TI "clinical") AND (TI manager* OR TI personnel* OR TI "staff" OR TI worker* OR TI provider*)) OR ((AB "health" OR AB "healthcare" OR AB "medical" OR AB "clinical") AND (AB manager* OR AB personnel* OR AB "staff" OR AB worker* OR AB provider*)) OR TI professional* OR AB professional* OR TI specialist* OR AB specialist* OR TI physician* OR AB physician* OR TI doctor* OR AB doctor* OR TI practitioner* OR AB practitioner* OR TI clinician* OR AB clinician* OR TI nurse* OR AB nurse* OR TI "nursing assistant*" OR AB "nursing assistant*" OR TI "midwife" OR AB "midwife" OR TI "midwives" OR AB "midwives" OR TI pharmacist* OR AB pharmacist* OR TI therapist* OR AB therapist* OR TI physiotherapist* OR AB physiotherapist* OR TI dentist* OR AB dentist* OR TI radiographer* OR AB radiographer* OR TI nutritionist* OR AB nutritionist* OR MH "Counselors+" OR TI mentor* OR AB mentor* ) OR ( MH "Mentorship" OR TI coach* OR AB coach* OR MH "Counseling+" OR TI counsel* OR AB counsel* OR MH "Patient Education+" OR MH "Communication+" OR TI communication* OR AB communication* OR TI telecoach* OR AB telecoach* OR TI telementor* OR AB telementor* )) AND ( MH ( randomized controlled trials OR double‐blind studies OR single‐blind studies OR random assignment OR pretest‐posttest design OR cluster sample ) OR TI ( randomised OR randomized ) OR AB random* OR TI trial OR ( (MH (sample size) AND AB (assigned OR allocated OR control)) ) OR MH ( placebos OR crossover design OR comparative studies ) OR AB ( (control W5 group) OR (cluster W3 RCT) OR PT (randomized controlled trial)) ) NOT ( ( MH animals+ OR MH (animal studies) OR TI (animal model*) ) NOT MH (human) ) |
| 16 | #15 AND LA english |
|  | ( MH "Chronic Disease+" OR (TI chronic* AND (TI disease* OR TI condition* OR TI "ill" OR TI illness*)) OR (AB chronic* AND (AB disease* OR AB condition* OR AB "ill" OR AB illness*)) OR MH "Diabetes Mellitus+" OR TI diabet* OR AB diabet* OR MH "Renal Insufficiency, Chronic+" OR ((TI "kidney" OR TI "renal") AND (TI failure* OR TI disease*)) OR ((AB "kidney" OR AB "renal") AND (AB failure* OR AB disease*)) OR MH "Heart Diseases+" OR TI cardi* OR AB cardi* OR TI heart* OR AB heart* OR TI coronary* OR AB coronary* OR TI angina* OR AB angina* OR TI myocard* OR AB myocard* OR TI ventricul* OR AB ventricul* OR TI atrioventricul* OR AB atrioventricul* OR TI pericard* OR AB pericard* OR TI atrial* OR AB atrial* OR TI endocardi* OR AB endocardi* OR TI arrhythmi* OR AB arrhythmi* OR TI thrombo* OR AB thrombo* OR TI tachy* OR AB tachy* OR TI brady* OR AB brady* OR TI fibrillat* OR AB fibrillat* OR MH "Stroke+" OR TI "stroke" OR AB "stroke" OR TI "strokes" OR AB "strokes" OR TI "cerebral vascular" OR AB "cerebral vascular" OR TI "cerebrovascular" OR AB "cerebrovascular" OR MH "Pulmonary Disease, Chronic Obstructive+" OR TI "COPD" OR AB "COPD" OR (TI obstruct* AND (TI "pulmonary" OR TI lung* OR TI airway* OR TI airflow* OR TI bronch* OR TI respirat*)) OR (AB obstruct* AND (AB "pulmonary" OR AB lung* OR AB airway* OR AB airflow* OR AB bronch* OR AB respirat*)) OR MH "Obesity+" OR TI obes* OR AB obes* OR TI overweight* OR AB overweight* OR TI "over weight*" OR AB "over weight*" OR MH "Metabolic Syndrome X+" ) AND ( TI "mhealth" OR AB "mhealth" OR TI "m health" OR AB "m health" OR TI "eHealth" OR AB "eHealth" OR TI "e health" OR AB "e health" OR TI "digital health" OR AB "digital health" OR MH "Digital Health+" OR MH "Telehealth+" OR MH "Mobile Applications" OR TI "mobile" OR AB "mobile" OR TI "mobiles" OR AB "mobiles" OR TI "app" OR AB "app" OR TI "apps" OR AB "apps" OR TI "Application" OR AB "Application" OR TI "Applications" OR AB "Applications" OR MH "Cellular Phone+" OR MH "Computers, Hand-Held+" OR TI smartphone* OR AB smartphone* OR TI "smart phone*" OR AB "smart phone*" OR TI "cellular phone*" OR AB "cellular phone*" OR TI "cell phone*" OR AB "cell phone*" OR TI "wearable" OR AB "wearable" OR TI smartwatch* OR AB smartwatch* OR TI "smart watch*" OR AB "smart watch*" OR TI tablet* OR AB tablet* ) AND (( MH "Health Personnel+" OR ((TI "health" OR TI "healthcare" OR TI "medical" OR TI "clinical") AND (TI manager* OR TI personnel* OR TI "staff" OR TI worker* OR TI provider*)) OR ((AB "health" OR AB "healthcare" OR AB "medical" OR AB "clinical") AND (AB manager* OR AB personnel* OR AB "staff" OR AB worker* OR AB provider*)) OR TI professional* OR AB professional* OR TI specialist* OR AB specialist* OR TI physician* OR AB physician* OR TI doctor* OR AB doctor* OR TI practitioner* OR AB practitioner* OR TI clinician* OR AB clinician* OR TI nurse* OR AB nurse* OR TI "nursing assistant*" OR AB "nursing assistant*" OR TI "midwife" OR AB "midwife" OR TI "midwives" OR AB "midwives" OR TI pharmacist* OR AB pharmacist* OR TI therapist* OR AB therapist* OR TI physiotherapist* OR AB physiotherapist* OR TI dentist* OR AB dentist* OR TI radiographer* OR AB radiographer* OR TI nutritionist* OR AB nutritionist* OR MH "Counselors+" OR TI mentor* OR AB mentor* ) OR ( MH "Mentorship" OR TI coach* OR AB coach* OR MH "Counseling+" OR TI counsel* OR AB counsel* OR MH "Patient Education+" OR MH "Communication+" OR TI communication* OR AB communication* OR TI telecoach* OR AB telecoach* OR TI telementor* OR AB telementor* )) AND ( MH ( randomized controlled trials OR double‐blind studies OR single‐blind studies OR random assignment OR pretest‐posttest design OR cluster sample ) OR TI ( randomised OR randomized ) OR AB random* OR TI trial OR ( (MH (sample size) AND AB (assigned OR allocated OR control)) ) OR MH ( placebos OR crossover design OR comparative studies ) OR AB ( (control W5 group) OR (cluster W3 RCT) OR PT (randomized controlled trial)) ) NOT ( ( MH animals+ OR MH (animal studies) OR TI (animal model*) ) NOT MH (human) ) AND LA english |
| 17 | #15 AND LA english AND MX Y |
|  | ( MH "Chronic Disease+" OR (TI chronic* AND (TI disease* OR TI condition* OR TI "ill" OR TI illness*)) OR (AB chronic* AND (AB disease* OR AB condition* OR AB "ill" OR AB illness*)) OR MH "Diabetes Mellitus+" OR TI diabet* OR AB diabet* OR MH "Renal Insufficiency, Chronic+" OR ((TI "kidney" OR TI "renal") AND (TI failure* OR TI disease*)) OR ((AB "kidney" OR AB "renal") AND (AB failure* OR AB disease*)) OR MH "Heart Diseases+" OR TI cardi* OR AB cardi* OR TI heart* OR AB heart* OR TI coronary* OR AB coronary* OR TI angina* OR AB angina* OR TI myocard* OR AB myocard* OR TI ventricul* OR AB ventricul* OR TI atrioventricul* OR AB atrioventricul* OR TI pericard* OR AB pericard* OR TI atrial* OR AB atrial* OR TI endocardi* OR AB endocardi* OR TI arrhythmi* OR AB arrhythmi* OR TI thrombo* OR AB thrombo* OR TI tachy* OR AB tachy* OR TI brady* OR AB brady* OR TI fibrillat* OR AB fibrillat* OR MH "Stroke+" OR TI "stroke" OR AB "stroke" OR TI "strokes" OR AB "strokes" OR TI "cerebral vascular" OR AB "cerebral vascular" OR TI "cerebrovascular" OR AB "cerebrovascular" OR MH "Pulmonary Disease, Chronic Obstructive+" OR TI "COPD" OR AB "COPD" OR (TI obstruct* AND (TI "pulmonary" OR TI lung* OR TI airway* OR TI airflow* OR TI bronch* OR TI respirat*)) OR (AB obstruct* AND (AB "pulmonary" OR AB lung* OR AB airway* OR AB airflow* OR AB bronch* OR AB respirat*)) OR MH "Obesity+" OR TI obes* OR AB obes* OR TI overweight* OR AB overweight* OR TI "over weight*" OR AB "over weight*" OR MH "Metabolic Syndrome X+" ) AND ( TI "mhealth" OR AB "mhealth" OR TI "m health" OR AB "m health" OR TI "eHealth" OR AB "eHealth" OR TI "e health" OR AB "e health" OR TI "digital health" OR AB "digital health" OR MH "Digital Health+" OR MH "Telehealth+" OR MH "Mobile Applications" OR TI "mobile" OR AB "mobile" OR TI "mobiles" OR AB "mobiles" OR TI "app" OR AB "app" OR TI "apps" OR AB "apps" OR TI "Application" OR AB "Application" OR TI "Applications" OR AB "Applications" OR MH "Cellular Phone+" OR MH "Computers, Hand-Held+" OR TI smartphone* OR AB smartphone* OR TI "smart phone*" OR AB "smart phone*" OR TI "cellular phone*" OR AB "cellular phone*" OR TI "cell phone*" OR AB "cell phone*" OR TI "wearable" OR AB "wearable" OR TI smartwatch* OR AB smartwatch* OR TI "smart watch*" OR AB "smart watch*" OR TI tablet* OR AB tablet* ) AND (( MH "Health Personnel+" OR ((TI "health" OR TI "healthcare" OR TI "medical" OR TI "clinical") AND (TI manager* OR TI personnel* OR TI "staff" OR TI worker* OR TI provider*)) OR ((AB "health" OR AB "healthcare" OR AB "medical" OR AB "clinical") AND (AB manager* OR AB personnel* OR AB "staff" OR AB worker* OR AB provider*)) OR TI professional* OR AB professional* OR TI specialist* OR AB specialist* OR TI physician* OR AB physician* OR TI doctor* OR AB doctor* OR TI practitioner* OR AB practitioner* OR TI clinician* OR AB clinician* OR TI nurse* OR AB nurse* OR TI "nursing assistant*" OR AB "nursing assistant*" OR TI "midwife" OR AB "midwife" OR TI "midwives" OR AB "midwives" OR TI pharmacist* OR AB pharmacist* OR TI therapist* OR AB therapist* OR TI physiotherapist* OR AB physiotherapist* OR TI dentist* OR AB dentist* OR TI radiographer* OR AB radiographer* OR TI nutritionist* OR AB nutritionist* OR MH "Counselors+" OR TI mentor* OR AB mentor* ) OR ( MH "Mentorship" OR TI coach* OR AB coach* OR MH "Counseling+" OR TI counsel* OR AB counsel* OR MH "Patient Education+" OR MH "Communication+" OR TI communication* OR AB communication* OR TI telecoach* OR AB telecoach* OR TI telementor* OR AB telementor* )) AND ( MH ( randomized controlled trials OR double‐blind studies OR single‐blind studies OR random assignment OR pretest‐posttest design OR cluster sample ) OR TI ( randomised OR randomized ) OR AB random* OR TI trial OR ( (MH (sample size) AND AB (assigned OR allocated OR control)) ) OR MH ( placebos OR crossover design OR comparative studies ) OR AB ( (control W5 group) OR (cluster W3 RCT) OR PT (randomized controlled trial)) ) NOT ( ( MH animals+ OR MH (animal studies) OR TI (animal model*) ) NOT MH (human) ) AND LA english AND MX Y |

Web of Science

| # | 検索式 |
| --- | --- |
| 1 | TS=(chronic* AND (disease* OR condition* OR ill OR illness*)) |
| 2 | TS=(diabet*) |
| 3 | TS=((kidney OR renal) AND (failure* OR disease*)) |
| 4 | TS=(cardi* OR heart* OR coronary* OR angina* OR myocard* OR ventricul* OR atrioventricul* OR pericard* OR atrial* OR endocardi* OR arrhythmi* OR thrombo* OR tachy* OR brady* OR fibrillat*) |
| 5 | TS=(stroke OR Strokes OR "Cerebral Vascular" OR cerebrovascular) |
| 6 | TS=(COPD OR ((obstruct*) AND (pulmonary OR lung* OR airway* OR airflow* OR bronch* OR respirat*))) |
| 7 | TS=(obes* OR overweight* OR "over weight*" OR "Metabolic Syndrome") |
| 8 | #1 OR #2 OR #3 OR #4 OR #5 OR #6 OR #7 |
|  | TS=((chronic* AND (disease* OR condition* OR ill OR illness*)) OR diabet* OR ((kidney OR renal) AND (failure* OR disease*)) OR cardi* OR heart* OR coronary* OR angina* OR myocard* OR ventricul* OR atrioventricul* OR pericard* OR atrial* OR endocardi* OR arrhythmi* OR thrombo* OR tachy* OR brady* OR fibrillat* OR stroke OR Strokes OR "Cerebral Vascular" OR cerebrovascular OR COPD OR ((obstruct*) AND (pulmonary OR lung* OR airway* OR airflow* OR bronch* OR respirat*)) OR obes* OR overweight* OR "over weight*" OR "Metabolic Syndrome") |
| 9 | TS=(mhealth OR "m health" OR eHealth OR "e health" OR "digital health" OR Telemedicine* OR mobile OR mobiles OR app OR apps OR Application OR Applications OR smartphone* OR "smart phone*" OR "cellular phone*" OR "cell phone*" OR wearable OR smartwatch* OR "smart watch*" OR tablet*) |
| 10 | TS=(((health OR healthcare OR medical OR clinical) AND (manager* OR personnel* OR staff OR worker* OR provider*)) OR professional* OR specialist* OR physician* OR doctor* OR practitioner* OR clinician* OR nurse* OR "nursing assistant*" OR midwife OR midwives OR pharmacist* OR physiotherapist* OR dentist* OR radiographer* OR Nutritionist* OR Counselor OR Counselors OR therapist*) |
| 11 | TS=(coach* OR mentor* OR counsel* OR communication* OR telecoach* OR telementor*) |
| 12 | #10 OR #11 |
|  | TS=(((health OR healthcare OR medical OR clinical) AND (manager* OR personnel* OR staff OR worker* OR provider*)) OR professional* OR specialist* OR physician* OR doctor* OR practitioner* OR clinician* OR nurse* OR "nursing assistant*" OR midwife OR midwives OR pharmacist* OR physiotherapist* OR dentist* OR radiographer* OR Nutritionist* OR Counselor OR Counselors OR therapist* OR coach* OR mentor* OR counsel* OR communication* OR telecoach* OR telementor*) |
| 13 | #8 AND #9 AND #12 |
|  | TS=(((chronic* AND (disease* OR condition* OR ill OR illness*)) OR diabet* OR ((kidney OR renal) AND (failure* OR disease*)) OR cardi* OR heart* OR coronary* OR angina* OR myocard* OR ventricul* OR atrioventricul* OR pericard* OR atrial* OR endocardi* OR arrhythmi* OR thrombo* OR tachy* OR brady* OR fibrillat* OR stroke OR Strokes OR "Cerebral Vascular" OR cerebrovascular OR COPD OR ((obstruct*) AND (pulmonary OR lung* OR airway* OR airflow* OR bronch* OR respirat*)) OR obes* OR overweight* OR "over weight*" OR "Metabolic Syndrome") AND (mhealth OR "m health" OR eHealth OR "e health" OR "digital health" OR Telemedicine* OR mobile OR mobiles OR app OR apps OR Application OR Applications OR smartphone* OR "smart phone*" OR "cellular phone*" OR "cell phone*" OR wearable OR smartwatch* OR "smart watch*" OR tablet*) AND (((health OR healthcare OR medical OR clinical) AND (manager* OR personnel* OR staff OR worker* OR provider*)) OR professional* OR specialist* OR physician* OR doctor* OR practitioner* OR clinician* OR nurse* OR "nursing assistant*" OR midwife OR midwives OR pharmacist* OR physiotherapist* OR dentist* OR radiographer* OR Nutritionist* OR Counselor OR Counselors OR therapist* OR coach* OR mentor* OR counsel* OR communication* OR telecoach* OR telementor*)) |
| 14 | #13 AND LA=(English) |
|  | TS=(((chronic* AND (disease* OR condition* OR ill OR illness*)) OR diabet* OR ((kidney OR renal) AND (failure* OR disease*)) OR cardi* OR heart* OR coronary* OR angina* OR myocard* OR ventricul* OR atrioventricul* OR pericard* OR atrial* OR endocardi* OR arrhythmi* OR thrombo* OR tachy* OR brady* OR fibrillat* OR stroke OR Strokes OR "Cerebral Vascular" OR cerebrovascular OR COPD OR ((obstruct*) AND (pulmonary OR lung* OR airway* OR airflow* OR bronch* OR respirat*)) OR obes* OR overweight* OR "over weight*" OR "Metabolic Syndrome") AND (mhealth OR "m health" OR eHealth OR "e health" OR "digital health" OR Telemedicine* OR mobile OR mobiles OR app OR apps OR Application OR Applications OR smartphone* OR "smart phone*" OR "cellular phone*" OR "cell phone*" OR wearable OR smartwatch* OR "smart watch*" OR tablet*) AND (((health OR healthcare OR medical OR clinical) AND (manager* OR personnel* OR staff OR worker* OR provider*)) OR professional* OR specialist* OR physician* OR doctor* OR practitioner* OR clinician* OR nurse* OR "nursing assistant*" OR midwife OR midwives OR pharmacist* OR physiotherapist* OR dentist* OR radiographer* OR Nutritionist* OR Counselor OR Counselors OR therapist* OR coach* OR mentor* OR counsel* OR communication* OR telecoach* OR telementor*)) AND LA=(English) |
| 15 | TS=(randomised OR randomized OR randomisation OR randomisation OR placebo* OR (random* AND (allocat* OR assign*)) OR (blind* AND (single OR double OR treble OR triple))) |
| 16 | #14 AND #15 |
|  | (TS=(((chronic* AND (disease* OR condition* OR ill OR illness*)) OR diabet* OR ((kidney OR renal) AND (failure* OR disease*)) OR cardi* OR heart* OR coronary* OR angina* OR myocard* OR ventricul* OR atrioventricul* OR pericard* OR atrial* OR endocardi* OR arrhythmi* OR thrombo* OR tachy* OR brady* OR fibrillat* OR stroke OR Strokes OR "Cerebral Vascular" OR cerebrovascular OR COPD OR ((obstruct*) AND (pulmonary OR lung* OR airway* OR airflow* OR bronch* OR respirat*)) OR obes* OR overweight* OR "over weight*" OR "Metabolic Syndrome") AND (mhealth OR "m health" OR eHealth OR "e health" OR "digital health" OR Telemedicine* OR mobile OR mobiles OR app OR apps OR Application OR Applications OR smartphone* OR "smart phone*" OR "cellular phone*" OR "cell phone*" OR wearable OR smartwatch* OR "smart watch*" OR tablet*) AND (((health OR healthcare OR medical OR clinical) AND (manager* OR personnel* OR staff OR worker* OR provider*)) OR professional* OR specialist* OR physician* OR doctor* OR practitioner* OR clinician* OR nurse* OR "nursing assistant*" OR midwife OR midwives OR pharmacist* OR physiotherapist* OR dentist* OR radiographer* OR Nutritionist* OR Counselor OR Counselors OR therapist* OR coach* OR mentor* OR counsel* OR communication* OR telecoach* OR telementor*)) AND LA=(English)) AND TS=(randomised OR randomized OR randomisation OR randomisation OR placebo* OR (random* AND (allocat* OR assign*)) OR (blind* AND (single OR double OR treble OR triple))) |
