# Supplementary material for: The Effect of Combining mHealth and Health Professional–Led Intervention for Improving Health-Related Outcomes in Chronic Diseases: Systematic Review and Meta-Analysis
Source: Interact J Med Res. 2025 Jan 20;14:e55835. doi: 10.2196/55835 (PMC11791457; doi:10.2196/55835)
Supplement: Multimedia Appendix 3 [file ijmr_v14i1e55835_app3.docx]

Table 1 Characteristics of included studies with author names beginning with A to Z~~

| Study and country | Population | sample size (n) | Intervention | Usual care | Outcomes | Key findings |
| --- | --- | --- | --- | --- | --- | --- |
| Alonso-Domínguez et al[., 2019, Spain](https://rayyan.ai/fulltexts/1651350) | Type 2 diabetes | n=204 (102/102) | Combined with a nurse-led food workshop, a smartphone app, and heart-healthy walking. Plus 10 minutes of standardized counseling on healthy eating and exercise. | Standardized counselling for 10 min about healthy eating and physical activity. | Physical Activity, Drug Use, Blood Pressure, BMI, Biochemical Parameters (Fasting Plasma Glucose, Glycated Hemoglobin, Triglycerides, Total Serum Cholesterol, LDL Cholesterol, HDL Cholesterol) | After 3 months, the intervention group increased daily steps, aerobic steps, and weekly metabolic minutes, while reducing sitting time. At 12 months, they maintained higher activity levels than the control group (P < 0.05). |
| [Anzaldo-Campos et al, 2016, Mexico](https://rayyan.ai/fulltexts/1651368) | Type 2 diabetes | n=201(102/99) | A combination of care management by a multidisciplinary team led by trained clinicians and nurses, and a peer-led group education component in addition to the technology-enabled intervention. | Only a combination of care management by a multidisciplinary team led by trained clinicians and nurses, and a peer-led group education component. | HbA1c, Total Cholesterol, LDL-c, HDL-c, Triglycerides, SBP, DBP, BMI, Self-efficacy, Depression, Lifestyle, Quality of Life, Diabetes Knowledge | Project Dulce significantly reduced HbA1c and improved diabetes knowledge in type 2 diabetes patients, with no major differences between tech-enhanced and standard programs compared to the control group. |
| [Araya et al., 2021, UK](https://rayyan.ai/fulltexts/1651375) | Depressive symptoms among people with diabetes and/or hypertension | n=1232 (617/615) | The digital intervention in the intervention group provided 18 short mini-sessions over a six-week period and was based on a behavioral activation approach. Nursing assistants provided participants with a smartphone at the first session and provided support via two mandatory phone calls. | Usual care, with treatment for depression, hypertension, or diabetes left to the discretion of local clinicians. They were assessed several times for depressive symptoms and referred to specialist services if at risk. | Depressive Symptoms Improvement, 6-Month PHQ-9 Score Reduction, Quality of Life, Disability, Behavioral Activation, Health Care Service Utilization | In São Paulo, 40.7% in the digital group and 28.6% in usual care showed a ≥50% PHQ-9 score reduction at 3 months, a significant 12.1% difference (P = .001). In Lima, this reduction was 52.7% vs. 34.1%, an 18.6% difference (P < .001). At 6 months, the differences were not significant. |
| [Azeltin et al., 2021, USA](https://rayyan.ai/fulltexts/1651408) | Type 2 diabetes | n=45 (25/20) | The intervention group underwent a 12-week digital health coaching program, including weekly phone visits and daily text messages for monitoring and support. Phone calls consisted of three elements: assessment of stages of change, review of weekly fasting glucose results, and setting of stage-appropriate goals. In addition, visual infographics were used to illustrate health goals, and daily blood glucose readings were recorded using the Healthy at Home remote patient monitoring system. | received care at the Family medicine residency clinic which included a physicians’ office visit at the beginning and end of the study period | Primary Outcomes: HbA1c, Fasting Blood Glucose, Fasting C-peptide Levels, HOMA2-%β, HOMA2-IR. Secondary Outcomes: Ethnicity, Exercise Vital Sign (EVS), Years Diagnosed with T2DM, Blood Pressure, Weight, ED Visits, Primary Care Office Visits, Changes to Medications, Adverse Events. | At 12 months, the intervention group showed a significant improvement in the Homeostatic Model Assessment for Insulin Resistance (HOMA2-IR), a marker of insulin resistance (p = 0.029). This significance remained even after adjusting for years since T2DM diagnosis, Medicaid enrollment, food access, baseline change stages, and race (p = 0.027). |
| [Blair et al., 2021, Mexico](https://rayyan.ai/fulltexts/1651430) | Cancer survivors | n=36 (18/18) | Educational materials, a wearable activity tracker, a free smartphone app, and 5 lines of support + additional health coaching to help you reach your study goals | Educational materials, wearable activity tracker called Jawbone, free smartphone app, and 5 phone support calls only | Primary Outcomes: Changes in Total Sedentary Time, Number of Breaks from Sitting, Break Ratio. Secondary Outcomes: Device-Based Measures of Sedentary Behavior and Physical Activity, Objectively Measured Physical Performance, Subjective Measures (Quality of Life, Pain, Fatigue). Other Measures: Sociodemographics, Cancer-Related Data, Comorbidities, Height and Weight, BMI. | The intervention group showed no significant changes in total sitting time, number of breaks, or prolonged sitting durations. However, a significant increase of 1675 steps per day, mainly of moderate intensity, was observed (P = .009). |
| [Blasco et al., 2012, Spain](https://rayyan.ai/fulltexts/1651431) | Acute coronary syndrome survivors | n=170 (87/83) | Patients were given an automatic sphygmomanometer, glucose and lipid meter, and a cellular phone to measure and send their blood pressure, heart rate, weight, glucose, and lipids on a scheduled basis. A cardiologist accessed this data through a secure web application and sent individualized recommendations via text messages during the 12-month follow-up period. | Same as intervention group without support person | Primary Outcome: Cardiovascular Risk Improvement.  Secondary Outcomes: Resting BP, BMI, Smoking Status, LDL-c, HbA1c, Treatment Goals Achievement, Quantitative Changes (in LDL-c, BP, BMI, HbA1c), Quality of Life and Anxiety Level. | After 12 months, the intervention group showed more improvement in cardiovascular risk profiles (P ≤ .010), better BP and HbA1c levels (P ≤ .012 and P ≤ .018, respectively), but no difference in smoking cessation or LDL-c. Their BMI was significantly lower (P ≤ .005) compared to the control group. |
| [Broers et al., 2020, Netherlands](https://rayyan.ai/fulltexts/1651450) | Hypertension, coronary artery disease or symptomatic heart failure | n=150 (76/74) | Group received ambulatory health-behavior assessment technologies for 6 months combined with a 3-month behavioral intervention program. | Group did not receive the behavioral　intervention prompts or the wearable and monitoring devices. Patients in this condition received the treatment as usual (e.g., regular outpatient hospital visits) | Primary Outcome Measures: Life-style. Secondary Outcome Measures: Quality of Life: | After the specified period, the intervention group showed significant improvement in lifestyle behaviors (p = .003), but no significant improvement in quality of life was observed (p = .447, p = .67). |
| [Chow et al., 2022, USA](https://rayyan.ai/fulltexts/1800807) | Cancer survivors | n=41 (24/17) | Participants tracked physical activity and diet using Fitbit and Healthwatch360, with smokers using the 'iCanQuit' app. The intervention group set weekly physical activity and diet goals, receiving updates through texts and emails, and accessed a Facebook group for support. Phone sessions with healthcare professionals focused on action plans for cardiovascular health and overcoming barriers, supplemented by motivational messages from research staff. | Group had access to the apps but received no reminders to use them, no coaching on goal setting and no feedback on activity or diet. | Primary Outcome Measures: Body Measurements, Blood Tests, Cardiopulmonary Reserve.  Secondary Outcome Measures: Cardiovascular Health History, Medication Adherence, Physical Activity Levels, Smoking History, Health-related Quality of Life, Health-related Self-efficacy, Behavior and Attitudes, Dietary Intake, Activity Monitoring. | The intervention group showed trends of improvement in dietary factors, healthy eating scores, time spent in moderate-to-high intensity physical activities, and daily step count, but these changes did not reach statistical significance. |
| [Coombes et al., 2021, australia](https://rayyan.ai/fulltexts/1810591) | Type 2 diabetes | n=30 (14/16) | The m-health program used a wrist-worn monitor and the PAI Health app to measure heart rate and provide feedback on PAI scores. The expert intervention consisted of four small-group sessions that included PAI concept education, brief guided exercises, and behavioral counseling. | Patients continued care with their regular GP and healthcare providers, adhered to exercise guidelines for T2D, received a brochure on exercise's role, and participated in sessions to improve balance and flexibility. | Primary Outcome Measures: Feasibility (Attendance, PAI Maintenance).  Secondary Outcome Measures: Efficacy (Glycemic Control, Cardiorespiratory Fitness, Exercise Capacity, Body Composition, Physical Activity, Sleep Duration, Health-Related Quality of Life), Acceptability (Participant Feedback), Safety (Adverse Events Monitoring). | The intervention group showed trends toward improvement in dietary factors, healthy eating scores, time spent in moderate-to-vigorous physical activity, and daily steps, but these changes did not reach statistical significance. |
| [Demeyer et al., 2017, UK](https://rayyan.ai/fulltexts/1810713) | Chronic obstructive pulmonary disease | n=343 (171/172) | The m-health intervention included Fitbug Air step counters, a smartphone app, and weekly goals with feedback. Professional support involved initial motivational interviews, home exercise booklets, weekly group texts, and phone calls for participants not complying or not progressing. | Patients received standard care from community physicians and cardiologists at discharge after hospitalization, including brief in-hospital health education by ward nurses, medication management, and follow-up visits with cardiologists or other providers based on self-assessment of cardiovascular health post-percutaneous coronary intervention. In the control group, WeChat was used solely for sending follow-up reminders. | Primary Outcome: Step Count Increase Over 3 Months. Secondary Outcomes: Moderate Physical Activity Duration, Walking Time and Intensity, Postbronchodilator Spirometry, Functional Exercise Capacity, Isometric Quadriceps Force, Health Status, Breathlessness. | After 12 weeks of intervention, the intervention group showed significant improvements in step count, time spent in moderate physical activity, and 6-minute walk distance. However, there were no significant differences in other health outcomes. |
| [Dorje et al., 2019, China](https://rayyan.ai/fulltexts/1810793) | Coronary heart disease | n=312 (156/156) | The m-health intervention offered cardiac rehabilitation and secondary prevention programs via the WeChat platform. This included activity tracking and monitoring of blood pressure and heart rate. The expert intervention involved individualized feedback and cardiac rehabilitation advice through WeChat consultations and regular reviews of health data. | Post-hospitalization, patients received standard care including nurse-provided health education, medication management, and cardiologist follow-ups based on self-assessed heart health after coronary intervention. The control group used WeChat only for follow-up reminders. | Primary Outcome: Functional Capacity Change.  Secondary Outcomes: Knowledge and Awareness of CHD, Resting Heart Rate, Systolic Blood Pressure, Cardiac Rehabilitation and Secondary Prevention Needs, Lipid Profile, Medication Adherence, Smoking Status, Obesity, Psychosocial Wellbeing, Quality of Life. Additional Outcomes: Fasting Plasma Glucose, Dietary Habits, Low Physical Activity Levels, Major Adverse Cardiac Events, SMART-CR/SP Programme-related Safety Issues. | Two months after the intervention, the intervention group showed a significant improvement in the 6-minute walk distance, with an increase of 20.64 meters. This improvement was maintained at 6 months, with an average difference of 22.29 meters between the intervention and control groups. |
| [Franc et al., 2020, Spain](https://rayyan.ai/fulltexts/1818343) | Type 1 and type 2 diabetes | n=665 (221/231/213) | The m-health intervention involved the DIABEO app, where patients entered data like blood sugar and activity, and the app calculated insulin doses. Expert intervention included setting targets and self-adjustment parameters. Nurses aided in managing blood sugar via the app, offering necessary feedback and advice. | The DIABEOsystem only, usual care only | Primary Outcome Measure: HbA1c Change.  Secondary Outcome Measures: DIABEO Usage Rates, Predictive Factors, Occurrence of Hypoglycemia, Severe Hypoglycemic Episode, Symptomatic Hypoglycemic Episode, Quality of Life. | The intervention group using DIABEO at least once daily showed a significant improvement in HbA1c levels at 12 months (P ≤ 0.001). However, there was no significant difference between the group using DIABEO alone and the group combining it with telemonitoring. |
| [Gill et al., 2019, Canada](https://rayyan.ai/fulltexts/1818345) | Risk factors for chronic disease | n=118 (59/59) | The m-health program used the HealtheSteps™ app for tracking health goals and virtual coaching, with an online social network for additional participant and coach interaction. The expert intervention consisted of regular sessions with trained coaches over six months, focusing on setting and achieving healthy lifestyle goals. | Continue with the　usual activities without restriction and provided public　available resources related to healthy lifestyles | Primary Outcome Measure: Average Number of Steps Per Day.  Secondary Outcome Measures: Total Physical Activity, Sedentary Time, Healthful Eating Score, Fatty Food Score, Fruit and Vegetable Consumption, Sugary Food Consumption, Self-rated Health, Weight, Body Mass Index, Waist Circumference, Cardiometabolic Measures. | After 6 months, the intervention group significantly increased their step count (p < 0.001), reduced their sedentary time (p = 0.03), and improved their overall healthy eating habits (p = 0.002). However, there were no between-group differences in self-reported physical activity, health-related quality of life, weight, waist circumference, or blood pressure at 6 months. |
| [Gonzalez-Sanchez et al., 2019, Spain](https://rayyan.ai/fulltexts/1818352) | Chronic Disease | n=833 (415/418) | The m-health 'Evident II' project app provided dietary and physical activity information, promoting the Mediterranean diet and a 10,000 steps per day goal to encourage active lifestyles. The expert intervention offered standardized counseling on physical activity and the Mediterranean diet. Physical activity recommendations aimed for 30 minutes 5 days a week or 20 minutes of intense activity 3 days a week, while dietary advice focused on key aspects of the Mediterranean diet during 15-minute individual visits. | Brief counseling on physical activity and the mediterranean diet without mobile application | Primary Outcome: Effect on CVRFs and CVR.  Secondary Outcomes: Systolic Blood Pressure, Cholesterol (Total and HDL), LDL Cholesterol, Diabetes Mellitus, Medication for Hypertension and Hyperlipidemia, Smoking History, CVR Estimation. | The intervention group did not observe any significant change in cardiovascular risk at 3 months post intervention. However, after 12 months, there were significant improvements in systolic and diastolic blood pressure, total cholesterol, and triglycerides, but no overall improvement in cardiovascular risk. |
| [Grady et al., 2017, UK](https://rayyan.ai/fulltexts/1818356) | Type 1 and type 2 diabetes | n=128 (62/66) | The meter + app group used both a meter and an app to monitor blood glucose. They performed self-monitoring and were instructed to review SMBG trends, patterns, and insights on the app at least weekly. In addition, they received biweekly text messages from healthcare providers with specific diabetes-related advice and suggested adjustments. Providers personalized color range indicators for each participant, explained their features, reviewed 14-day app progress reports to generate diabetes-related texts, and regularly discussed progress with participants. | Blood pressure monitor monitoring only | Change in HbA1c | The intervention group showed a significant decrease in HbA1c compared to baseline at 12 and 24 weeks post intervention (p<.001). However, the difference in HbA1c reduction between the two groups was not statistically significant at 12 or 24 weeks. |
| [Haufe, et al., 2019, Germany](https://rayyan.ai/fulltexts/1818375) | Metabolic syndrome | n=314 (160/154) | The goal was 150 minutes of physical activity per week, with individualized suggestions for exercise via in-person meetings and a smartphone app. | Continued their current lifestyle | Change in Metabolic Syndrome Z Score, Work Ability, Exercise Capacity, Health-Related Quality of Life, Body Composition, Adherence to the Intervention | After a 6-month exercise intervention, the intervention group showed a significant decrease in the metabolic syndrome Z-score. The difference between the groups was -0.26 (95% CI: -0.35 to -0.16, p<0.01). |
| [Hisam et al., 2022, Pakistan](https://rayyan.ai/fulltexts/1705903) | Patients with post-acute coronary syndrome | n=160 (80/80) | The MCard intervention, which a cardiac rehabilitation program that supplements standard post-ACS care, including individualized psychotherapy during hospitalization and daily mobile text messages promoting healthy lifestyle changes via a dedicated app | Receives only standard post-ACS care | Health-Related Quality of Life (HRQoL) | Mean SF-12 physical component scores improved significantly in the MCard group at 12 and 24 weeks; mean SF-12 mental item scores also improved significantly in the MCard group at 12 and 24 weeks (p<.001); all domains of the MacNew QLMI (social, emotional, physical, and global) improved statistically significantly in the MCard group at 12 and 24 weeks (p<.001). |
| [Jiang et al., 2021, Singapore](https://rayyan.ai/fulltexts/1850957) | Heart failure | n=213 (70/71/72) | The m-health intervention used a smartphone app offering educational content, medication and appointment reminders, health logs, and chat access to a research nurse. Nurses also monitored patient symptoms via a web portal. The expert intervention was a nurse-led, 6-week home-based heart failure self-management program, featuring an education and self-management toolkit, psychosocial education, and bi-weekly home visits by the nurse to enhance patient self-care skills. | Only nurse-led psychosocial education approach without app, or control group | Primary Outcome: Heart Failure Self-Care.  Secondary Outcomes: Cardiac Self-Efficacy, Anxiety and Depression, Health-Related Quality of Life, Perceived Social Support, Clinical Outcomes (NYHA Classification, Unplanned Health Service Use). | At 6 weeks, 3 months, and 6 months, intervention groups A and B exhibited significantly better heart failure self-care, confidence, and management than the control group (p<0.05, p=0.001, p<0.001). Group B (m-health + app) also had fewer heart-related unplanned hospital visits and ER admissions at 6 months compared to controls, but no significant differences were found between groups A and B. |
| [Jiang et al., 2022, Singapore](https://rayyan.ai/fulltexts/1850971) | Type 2 diabetes | n=114 (58/56) | The m-health intervention involved a smartphone app serving as an educational resource and self-management tool for diabetes patients, enabling them to monitor and log blood sugar, diet, and exercise routines. The expert intervention included nurses providing personalized care and counseling to participants through scheduled calls and outpatient visits. | Regular in-person educational sessions and telephone follow-up at the diabetes outpatient clinic | Primary Outcome: Self-Efficacy.  Secondary Outcomes: Diabetes Self-Care, Health-Related Quality of Life, Acute Diabetes Complications, Unplanned Medical Consultations, HbA1c Levels. | The intervention group had lower HbA1c levels at the 6-month follow-up, but there was no statistically significant difference between the intervention and control groups. There was also no statistically significant difference between the two groups in the number of unplanned medical visits at the 6-month follow-up. |
| [Khunti et al., 2021, UK](https://rayyan.ai/fulltexts/1856291) | Diabetes | n=1366 (456/450/460) | m-health provided text messages encouraging pedometer use and tailored feedback on achieving step count goals. As part of the professional intervention, participants were called to set step count goals and action plans for the next six months. | Information leaflet | Primary Outcome: Ambulatory Activity (Steps/Day).  Secondary Outcomes: Sedentary Time, Light-Intensity Activity, Moderate- to Vigorous-Intensity Activity, Sitting/Lying Time, Standing Time, Walking Time, Physical Activity Level, Intervention Process Measures, HbA1C and Other Clinical Variables, Cost-Effectiveness, Quality-Adjusted Life-Year. | At 12 months, the "Walking Away Plus" group increased daily steps by 547 compared to controls and was 1.61 times more likely to achieve 150 minutes of moderate to strenuous activity. However, at 48 months, no significant differences in walking activity were noted between the groups. The "Walking Away" group showed no significant differences from the control at both 12 and 48 months. |
| [Manzoor et al., 2021, Pakistan](https://rayyan.ai/fulltexts/1856349) | Post-acute coronary syndrome | n=160 (80/80) | m-health used a specially created app to provide standardized messages about daily healthy lifestyle changes as mobile text during the day. It also included features to help track specific symptoms and activities. Specialist intervention provided a medically monitored cardiac rehabilitation program called MCard during hospitalization. Individual counseling was included. | Standard post-ACS treatment | Primary Outcomes: Physical Activity Level, Dietary Habits.  Secondary Outcomes: Smoking Status, Salt Intake, Walking Duration, Medication Adherence, BP and Weight Monitoring. | At 12 weeks, the intervention group showed significant improvement in healthy eating (p<0.001), and at 24 weeks, they improved in physical activity MET scores and healthy eating (p=0.007, p<0.001), with notable differences in salt intake and diet (p=0.008, p=0.012). No significant changes were observed in medication adherence, smoking, or self-monitoring of blood pressure and weight. |
| [Pamungkas et al., 2022, Indonesia](https://rayyan.ai/fulltexts/1856391) | Type 2 diabetes | n=60 (30/30) | The m-Health intervention featured a diabetes coaching smartphone app, providing healthy food choices, dietary portions, exercise recommendations, medication tips, and DSM practice guidance, including blood glucose monitoring over 12 weeks. Participants were encouraged to log their self-management activities in the app, supplemented by Zoom meetings and phone consultations. Professional interventions involved guiding patients, monitoring progress, addressing complications, and resolving challenges through online and phone interactions. | Routine services by local health centers | Primary Outcome: Self-management Practices.  Secondary Outcomes: HbA1c, Systolic and Diastolic Blood Pressure, HDL Level, LDL Level. | The intervention group demonstrated significantly better self-management behaviors such as dietary control, exercise, blood glucose monitoring, medication adherence, and post-intervention screening for complications than the control group. Clinical outcomes were also significantly better in the intervention group than in the control group. |
| [Sun et al., 2019, China](https://rayyan.ai/fulltexts/1856443) | Type 2 diabetes | n=91 (44/47) | The m-Health intervention involved patients uploading blood glucose data via an app, with the medical team sending advice and reminders bi-weekly. Patients also logged diet and activity data, receiving monthly dietary recommendations from a dietitian based on this input. The expert intervention focused on individualized dietary advice tailored to each patient's blood glucose levels. | Free glucometers were given out, and in-person follow-up was provided every three months to receive diet and exercise guidance. | Primary Outcomes: HbA1c Levels, Postprandial Blood Glucose Levels.  Secondary Outcomes: Age, Gender, Diabetes Duration, Fasting Blood Glucose, Total Cholesterol, Triglyceride Levels, HDL-C, LDL-C, Blood Urea Nitrogen, Creatinine Levels, AST Levels, ALT Levels, γ-Glutamyltransferase Levels, Body Mass Index, Systolic Blood Pressure, Diastolic Blood Pressure, Patient Satisfaction. | The intervention group showed significant improvement in postprandial blood glucose levels at 3 months postintervention (p<.05). In addition, after 6 months, postprandial blood glucose and glycated hemoglobin levels showed a significant decreasing trend compared to baseline and the control group (p<.05). |
| [Wong et al., 2022, China](https://rayyan.ai/fulltexts/1856507) | Coronary heart disease | n=60 (30/30) | The m-Health intervention utilized a CHD app to provide structured e-education content and support features. Participants entered their exercise time and blood pressure, receiving alerts for abnormal health data and regular motivational messages for exercise continuation. Expert intervention included briefings and feedback on abnormal data from trained research nurses. | Regular "participants are asked to quit smoking, take prescribed medications, and lead healthy lifestyles" telephone interventions by nurses | Primary Outcome: Total Amount of Physical Exercise.  Secondary Outcomes: Self-Efficacy in Illness Management, Clinical and Cardiovascular Risk Factors Profile. | The intervention group significantly increased in physical activity and significantly decreased in lipid concentrations (total cholesterol d = -0.43, triglycerides d = -0.39) at the end of the study. |
| [Yingyaun et al., 2022, Thiland](https://he02.tci-thaijo.org/index.php/PRIJNR/article/view/258070/175798) | Diabetes | n=80 (27/26/27) | The m-Health intervention used a mobile messenger app to deliver educational messages and images about insulin injection, covering insulin storage, injection techniques, and identifying lipohypertrophy. The expert intervention included face-to-face individual educational sessions, focusing on insulin injection practices, practical demonstrations, and information about insulin types and their effects. | Routine care only | Primary Outcome: Change in Hemoglobin A1c (HbA1c).  Secondary Outcomes: Change in Fasting Plasma Glucose (FPG), Change in Knowledge Scores on Insulin Injection. | After 6 months, both intervention groups 2 (m-health + health professional education) and 1 (only education) showed a significant reduction in hemoglobin A1c compared to the control group, but no significant difference between the two intervention groups. Knowledge scores about insulin injection in group 2 were significantly higher than both the control and group 1. No significant difference was found in blood glucose control related to education sessions. |
| [Zhang et al., 2019, China](https://rayyan.ai/fulltexts/1856517) | Diabetes | n=234 (78/78/78) | The m-Health intervention utilized the Welltang smartphone app for diabetes management, allowing patients to learn about diabetes, and track SMBG, diet, exercise, medication, and weight, while also enabling communication with clinicians. The expert intervention, delivered by a specialized diabetes care team via Welltang, provided comprehensive support including dietary guidance, exercise recommendations, blood sugar monitoring, and diabetes education based on patient data. | Usual care | Primary Outcome Measures: Body Mass Index (BMI), Blood Pressure, Waist Circumference.  Secondary Outcome Measures: Fasting Plasma Glucose (FPG), HbA1c, Total Cholesterol (TC), Triglyceride (TG), High-Density Lipoprotein Cholesterol (HDL-c), Low-Density Lipoprotein Cholesterol (LDL-c), Alanine Aminotransferase, Aspartate Aminotransferase, Gamma-Glutamyl Transpeptidase, Creatinine, Uric Acid, Albumin-to-Creatinine Ratio. | After 3 and 6 months, the intervention group significantly reduced HbA1c levels (p<.05). Notably, at 6 months, the interactive management app group had lower HbA1c than the self-management app group (p=.04). This group also showed greater HbA1c reduction compared to both the self-management app and control groups at both 3 and 6 months (p<.05). |
| [Wong et al., 2022, China](https://rayyan.ai/fulltexts/1856507) | Coronary heart disease | n=60 (30/30) | The m-Health intervention utilized a CHD app to provide structured e-education content and support features. Participants entered their exercise time and blood pressure, receiving alerts for abnormal health data and regular motivational messages for exercise continuation. Expert intervention included briefings and feedback on abnormal data from trained research nurses. | Regular "participants are asked to quit smoking, take prescribed medications, and lead healthy lifestyles" telephone interventions by nurses | Primary Outcome: Total Amount of Physical Exercise.  Secondary Outcomes: Self-Efficacy in Illness Management, Clinical and Cardiovascular Risk Factors Profile. | The intervention group significantly increased in physical activity and significantly decreased in lipid concentrations (total cholesterol d = -0.43, triglycerides d = -0.39) at the end of the study. |
|  |  |  |  |  |  |  |
