# Supplementary material for: The Effect of Combining mHealth and Health Professional–Led Intervention for Improving Health-Related Outcomes in Chronic Diseases: Systematic Review and Meta-Analysis
Source: Interact J Med Res. 2025 Jan 20;14:e55835. doi: 10.2196/55835 (PMC11791457; doi:10.2196/55835)
Supplement: Multimedia Appendix 4 [file ijmr_v14i1e55835_app4.docx]

**a. Risk of bias for each studies with HbA1c**

**b. Risk of bias for each studies with Quality of Life**

**c. Risk of bias for each studies with physical activity (steps)**

**d. Risk of bias for each studies with physical activity (subjective)**
