# Supplementary material for: The Effect of Combining mHealth and Health Professional–Led Intervention for Improving Health-Related Outcomes in Chronic Diseases: Systematic Review and Meta-Analysis
Source: Interact J Med Res. 2025 Jan 20;14:e55835. doi: 10.2196/55835 (PMC11791457; doi:10.2196/55835)
Supplement: Multimedia Appendix 5 [file ijmr_v14i1e55835_app5.docx]

**Appendix 2 For the quality of the clinical evidence, GRADE (Grading of Recommendations Assessment, Development, and Evaluation system)**

| **Certainty assessment** | | | | | | | **№ of patients** | | **Effect** | | **Certainty** | **Importance** |
| --- | --- | --- | --- | --- | --- | --- | --- | --- | --- | --- | --- | --- |
| **№ of studies** | **Study design** | **Risk of bias** | **Inconsistency** | **Indirectness** | **Imprecision** | **Other considerations** | **mhealth** | **control for HbA1c** | **Relative (95% CI)** | **Absolute (95% CI)** |  |  |
| **HbA1c for the short terms** | | | | | | | | | | | | |
| 9 | randomised trials | serious^a^ | very serious^b^ | not serious | not serious | none | 800 | 805 | - | SMD **0.43 lower** (0.64 lower to 0.21 lower) | ⨁◯◯◯ Very low |  |
| **HbA1c for the medium terms** | | | | | | | | | | | | |
| 5 | randomised trials | serious^a^ | not serious | not serious | not serious | none | 254 | 259 | - | SMD **0.29 lower** (0.49 lower to 0.09 lower) | ⨁⨁⨁◯ Moderate |  |
| **HbA1c for the long terms** | | | | | | | | | | | | |
| 4 | randomised trials | not serious | very serious^b^ | not serious | serious^c^ | none | 1070 | 1124 | - | SMD **0.23 lower** (0.49 lower to 0.03 higher) | ⨁◯◯◯ Very low |  |
| **Quality of Life (QOL) for short terms** | | | | | | | | | | | | |
| 8 | randomised trials | serious^a^ | serious^d^ | not serious | not serious | none | 922 | 915 | - | SMD **0.23 lower** (0.42 lower to 0.05 lower) | ⨁⨁◯◯ Low |  |
| **Quality of Life (QOL) for medium terms** | | | | | | | | | | | | |
| 8 | randomised trials | serious^a^ | not serious | not serious | not serious | none | 1139 | 1113 | - | SMD **0.16 lower** (0.24 lower to 0.07 lower) | ⨁⨁⨁◯ Moderate |  |
| **Quality of Life (QOL) for long terms** | | | | | | | | | | | | |
| 2 | randomised trials | serious^a^ | serious^d^ | not serious | serious^c^ | none | 448 | 496 | - | SMD **0.12 lower** (0.41 lower to 0.16 higher) | ⨁◯◯◯ Very low |  |
| **Physical Activity (Steps) for the short terms** | | | | | | | | | | | | |
| 4 | randomised trials | not serious | not serious | not serious | serious^c^ | none | 306 | 298 | - | SMD **0.11 higher** (0.05 lower to 0.27 higher) | ⨁⨁⨁◯ Moderate |  |
| **Physical Activity (Subjective) for the short terms** | | | | | | | | | | | | |
| 3 | randomised trials | serious^a^ | not serious | not serious | not serious | none | 212 | 212 | - | SMD **0.31 higher** (0.12 higher to 0.5 higher) | ⨁⨁⨁◯ Moderate |  |
| **Physical Activity (Subjective) for the medium terms** | | | | | | | | | | | | |
| 2 | randomised trials | serious^a^ | serious^d^ | not serious | serious^c^ | none | 139 | 139 | - | SMD **0.26 higher** (0.17 lower to 0.69 higher) | ⨁◯◯◯ Very low |  |
| **Physical Activity (Subjective) for the long terms** | | | | | | | | | | | | |
| 2 | randomised trials | serious^a^ | not serious | not serious | serious^c^ | none | 447 | 499 | - | SMD **0.15 higher** (0.05 lower to 0.36 higher) | ⨁⨁◯◯ Low |  |

**CI:** confidence interval; **SMD:** standardised mean difference

#### Explanations

a. Downgraded one level due to moderate risk of bias

b. Downgraded two levels due to I^2^>75%

c. due to no significant difference, rate down one level

d. Downgraded one level due to I^2^>50%
